# Identifying factors that may influence the classification performance of radiomics models using contrast-enhanced mammography (CEM) images

**DOI:** 10.1186/s40644-022-00460-8

**Published:** 2022-05-12

**Authors:** Yuqi Sun, Simin Wang, Ziang Liu, Chao You, Ruimin Li, Ning Mao, Shaofeng Duan, Henry S. Lynn, Yajia Gu

**Affiliations:** 1 Department of Biostatistics, Key Laboratory on Public Health Safety of the Ministry of Education, School of Public Health, Fudan University, Shanghai, 200032 China; 2 Department of Radiology, Fudan University Shanghai Cancer Center, No. 270 Dongan Road, Shanghai, 200032 China; 3 Department of Oncology, Shanghai Medical College, Fudan University, No. 270 Dongan Road, Shanghai, 200032 China; 4 Department of Biostatistics, School of Public Health, Yale University, New Haven, CT USA; 5 Department of Radiology, Yantai Yuhuangding Hospital, Qingdao University, Shandong, 264000 China; 6 GE Healthcare China, No. 1 Huatuo Road, Shanghai, 210000 China

**Keywords:** Mammography, Breast Cancer, Radiomics, Artifact

## Abstract

**Background:**

Radiomics plays an important role in the field of oncology. Few studies have focused on the identification of factors that may influence the classification performance of radiomics models. The goal of this study was to use contrast-enhanced mammography (CEM) images to identify factors that may potentially influence the performance of radiomics models in diagnosing breast lesions.

**Methods:**

A total of 157 women with 161 breast lesions were included. Least absolute shrinkage and selection operator (LASSO) regression and the random forest (RF) algorithm were employed to construct radiomics models. The classification result for each lesion was obtained by using 100 rounds of five-fold cross-validation. The image features interpreted by the radiologists were used in the exploratory factor analyses. Univariate and multivariate analyses were performed to determine the association between the image features and misclassification. Additional exploratory analyses were performed to examine the findings.

**Results:**

Among the lesions misclassified by both LASSO and RF ≥ 20% of the iterations in the cross-validation and those misclassified by both algorithms ≤5% of the iterations, univariate analysis showed that larger lesion size and the presence of rim artifacts and/or ripple artifacts were associated with more misclassifications among benign lesions, and smaller lesion size was associated with more misclassifications among malignant lesions (all *p* <  0.050). Multivariate analysis showed that smaller lesion size (odds ratio [OR] = 0.699, *p* = 0.002) and the presence of air trapping artifacts (OR = 35.568, *p* = 0.025) were factors that may lead to misclassification among malignant lesions. Additional exploratory analyses showed that benign lesions with rim artifacts and small malignant lesions (< 20 mm) with air trapping artifacts were misclassified by approximately 50% more in rate compared with benign and malignant lesions without these factors.

**Conclusions:**

Lesion size and artifacts in CEM images may affect the diagnostic performance of radiomics models. The classification results for lesions presenting with certain factors may be less reliable.

**Supplementary Information:**

The online version contains supplementary material available at 10.1186/s40644-022-00460-8.

## Introduction

It is important to find an accurate and efficient way to detect and diagnose breast cancer. In recent years, radiomics has played an increasingly important role in the field of oncology [1–4]. In radiomics, a high-throughput computer algorithm extracts large amounts of image features and converts medical images into quantitative data, showing decent results [5–7]. For breast cancer, radiomics has been extensively studied in research settings for diagnosis, treatment evaluation, and prognosis prediction [1–4, 8].

Contrast-enhanced mammography (CEM) is a technique that can simultaneously show the morphological and angiogenic characteristics of breast lesions [9, 10] and has a high spatial resolution comparable to that of conventional mammography [11, 12]. Several studies have developed and validated radiomics models in an attempt to achieve high diagnostic accuracy for breast lesions [13–18]. Although the diagnostic performance of radiomics models is promising, concerns still persist, as radiomics approaches are often regarded as black boxes and are less acceptable for clinical application [1, 2, 19, 20]. In other words, improvement in the overall diagnostic performance of radiomics models is still difficult to convert into practical clinical benefits, such as a reduction in unnecessary biopsies. Radiomics models are still not sufficiently reliable and interpretable to be used in the real-world diagnostic setting. In addition, few studies have examined imaging factors that may influence the diagnostic performance of the models.

The purpose of this study was to examine the performance of radiomics analysis in breast cancer diagnosis and preliminarily disentangle the black box of radiomics by identifying factors that may influence the classification results of radiomics models. Our study focused on breast lesions that were more likely to be misclassified by radiomics analysis and attempted to identify the potential image features that may influence the classification results from an interpretable perspective.

## Materials and methods

### Study participants

**Fig. 1:**
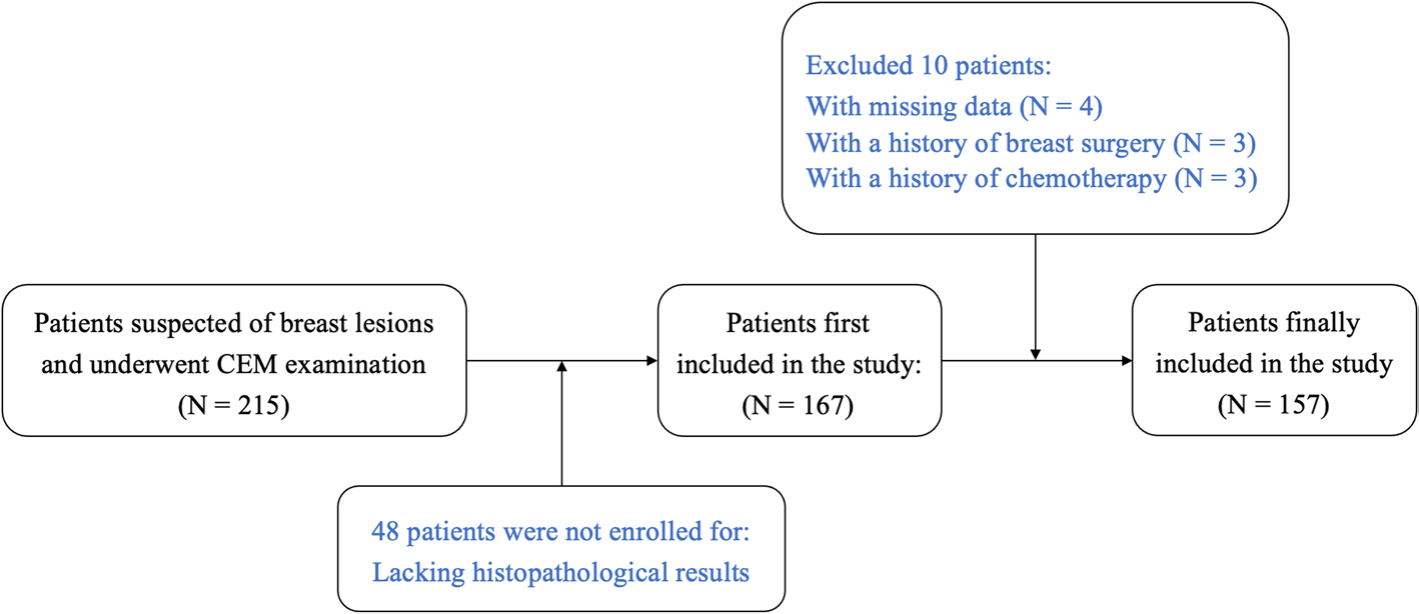
Patient inclusion and exclusion flowchart. CEM = contrast-enhanced mammography

This retrospective study was approved by the Institutional Review Board and Ethics Committee of the research center. The requirement for patient informed consent was waived. We collected consecutive CEM images between November 2018 and February 2020. The indications for CEM in this study included (1) problem solving for inconclusive findings on mammography or ultrasound screening; and (2) evaluation of symptomatic patients. The inclusion criteria were as follows: (1) patients with suspected breast lesions after physical examination or screening; (2) patients with referral for CEM by breast surgeons as part of diagnostic imaging; and (3) patients with final diagnoses that were confirmed by histopathological results. We excluded patients (1) with missing data and (2) with a history of breast surgery, breast radiotherapy, chemotherapy, or hormone treatment within 6 months prior to CEM examination. The patient inclusion and exclusion workflows are shown in Fig. 1. A total of 157 women with 161 breast lesions (47 benign, 29.2%; 114 malignant, 70.8%) were included in the study. The median age of the patients was 49 years (range, 21–70 years).

### CEM examination

All CEM examinations were performed using Senographe Essential mammography units (GE Healthcare). Before the examination, a dose of 1.5 mL/kg body weight iodinated contrast material (Iohexol, 300–350 mg I/mL) was injected intravenously using an automated power injector at a flow rate of 3.0 mL/s, followed by a 10-mL bolus of saline. Two minutes after the injection, bilateral craniocaudal (CC) views were obtained first, beginning with the suspicious breast. Then, bilateral mediolateral oblique (MLO) views were acquired in the same order. In a single projection, a pair of low-energy (LE) and high-energy (HE) exposures was performed within 1.5 seconds. The HE and LE images were recombined to generate dual-energy subtraction (DES) images. All of the HE, LE, and DES images were used to construct the radiomics models.

### CEM image evaluation

Two radiologists with 5–10 years of experience in breast imaging reviewed and interpreted all of the CEM images to obtain the image features. The radiologists were blinded to the histopathology results. When a discrepancy occurred in image evaluation, the final decision was made by consensus. The image features could be divided into two main groups: (1) basic image features and (2) artifact features. The basic image features included breast density, degree of background parenchymal enhancement (BPE), and lesion size. Breast density (a, b, c, or d) was evaluated using the LE images according to the Breast Imaging Reporting and Data System (BI-RADS) mammography lexicon [21]. The degree of BPE (minimal, mild, moderate, or marked) was assessed using the DES images referring to the BI-RADS MRI lexicon [22]. Lesion size was obtained by calculating the mean value of the largest lesion diameters on DES images measured by two independent radiologists. The artifact features included the presence of rim artifacts, ripple artifacts, vascular artifacts, and air trapping artifacts in DES images, as these artifacts occurred more often and might interfere with image quality [23, 24]. We defined artifacts located outside the lesion area as being absent since all the radiomics features were extracted from inside the lesion area and therefore might not interfere with artifacts outside the lesion area.

In addition, we extracted three objective quantitative features that might reflect the enhancement degree of the lesions. These features include the signal-to-noise ratio (SNR), contrast-to-noise ratio (CNR), and background contrast ratio (BCR). Since these features are obtained through calculation and can also be affected by the abovementioned image features, such as artifacts, they were excluded in the factor analysis. We only examined the distribution pattern of these features among the lesions with different classification results. The detailed processes and calculation methods of these features are provided in the Supplemental Materials (Appendix E1).

### Lesion delineation and feature extraction

The lesion contours were manually delineated with ITK-SNAP (version 3.6; www.itksnap.org) (25) by two radiologists together. For each lesion, a total of 6 regions of interest (ROIs) were delineated on the HE, LE, and DES images in the CC and MLO views. For multiple lesions within one breast, only the largest lesion was delineated.

Because the voxel was isotropic in-plane, we omitted the image resampling step. Gray-level discretization was performed to discretize all the images to 256 Gy levels. Spectral Mammography Kit (SMK) software (version 1.2.0, GE Healthcare) was used to extract the radiomics features. For each ROI, a total of 680 features, including 14 shape features, 18 first-order features, 24 Gy-level cooccurrence matrix (GLCM) features, 16 Gy-level run length matrix (GLRLM) features, 16 Gy-level size zone matrix (GLSZM) features, and 592 wavelet features, were extracted (Supplemental Table 1).

### Statistical analysis

#### Feature selection and Radiomics model building

We employed two algorithms, L1-based least absolute shrinkage and selection operator (LASSO) regression [25] and the random forest (RF) algorithm [26], with all the radiomics features (680 features for each ROI), to construct the classification models. The “one-standard-error” rule [27] was used to select the best model when implementing LASSO regression. The reference standard of the classification results was the histopathological results. To obtain robust results regarding how the radiomics models classified each lesion, we conducted 100 rounds of five-fold cross-validation. During each round of cross-validation, to account for imbalanced class numbers between malignant and benign lesions, adjusted weights inversely proportional to the frequencies of each class in the training data were calculated and incorporated in building RF and LASSO regression [28–30]. Before analysis, all the extracted radiomics features were normalized. We performed the feature normalization using the training data and calculated the mean and standard deviation for each feature. Subsequently, the values of mean and standard deviation were used to normalize the features in the testing data. Besides, the dimensions of radiomics features were reduced using the training data (80% of the whole data). We removed highly correlated redundant radiomics features if the pairwise correlations were greater than 0.8. Specifically, if two radiomics features had a correlation greater than 0.8, the radiomics feature with the largest mean absolute correlation was removed. Then, the models were built on the remaining features in the training data and the classification results for the testing data determined by using the best cutoff value based on the Youden index [31] for both the LASSO and RF models were summarized. The area under the curve (AUC), accuracy, sensitivity, and specificity values in the testing dataset were calculated. The misclassification probability for each lesion was obtained. The details of this statistical procedure are provided in the Supplemental Materials (Appendix E2).

#### Definition of lesion with high/low misclassification probability

For both the LASSO and RF method, we defined a lesion as having a high misclassification probability if it was incorrectly classified for no less than 20.0% of 100 iterations and as having a low misclassification probability if it was incorrectly classified for no more than 5.0% of the iterations. To combine the results of the LASSO regression and RF models, we defined a lesion as having a high misclassification probability for both algorithms if the lesion was defined with a high misclassification probability by each algorithm at the same time; the equivalent definition was used to identify lesions with a low misclassification probability for both algorithms. Unless otherwise specified, lesions described below as having a high/low misclassification probability are those with a high/low misclassification probability as determined by both algorithms simultaneously.

#### Identification of factors influencing the classification performance of Radiomics models

Multivariate logistic regression was conducted using the type of lesion (high misclassification probability vs. low misclassification probability) as a dependent variable and the image features as independent variables. A factor that showed a statistically significant high or low odds ratio (OR) was determined as an influential factor.

#### Additional exploratory analyses

To directly evaluate how the factors identified in the previous analysis influence the performances of the radiomics models, we compared the correct classification rates between lesions with certain factors and the lesions without these factors based on the results of cross-validation.

In addition, to evaluate the performance of radiomics models on the data with/without influential factors, we performed two more sets of 100 rounds of five-fold cross-validation with both radiomics algorithms built on the data, including the lesions with/without the factors identified by the factor analysis. The AUC, accuracy, sensitivity, and specificity values in the testing dataset were calculated for comparison.

#### General statistical analysis

Continuous variables were described as the means ± standard deviations, and categorical variables were summarized as proportions (%). Independent t tests, Wilcoxon rank-sum tests, and Fisher’s exact tests were used as appropriate for the univariate analyses and additional exploratory analyses. A *p* value less than 0.050 was considered statistically significant. All analyses were implemented in R software (version 3.6.3) [32].

## Results

### Summary of the study cohort and image features

A summary of the study cohort and image features is shown in Table 1. The mean age and lesion size in the malignant lesion group were significantly greater than those in the benign lesion group (*p* <  0.050). The distributions of different types of breast densities and degrees of BPE were significantly different between the two groups (both *p* <  0.050). For the different kinds of artifacts, significant differences were observed in the presence of ripple artifacts (*p* = 0.005) and vascular artifacts (*p* = 0.042), but no differences were found in the presence of rim artifacts (*p* = 1.000) and air trapping artifacts (*p* = 0.104) between the two groups. For the objective quantitative features, the benign lesion group showed lower SNR (*p* <  0.001), CNR (*p* <  0.001), and BCR values (*p* <  0.001) than the malignant lesion group.

**Table 1:** Summary of the study cohort and image features

Characteristics	Benign lesions (*n* = 47)	Malignant lesions (*n* = 114)	*P* value
Age (year) ^a^	46.0 ± 7.9	50.7 ± 9.2	0.002
Lesion size (mm) ^a^	17.1 ± 10.3	28.8 ± 15.6	< 0.001
Breast density			0.004
a-b	4/47 (8.5)	33/114 (28.9)	
c-d	43/47 (91.5)	81/114 (71.1)	
Degree of BPE			< 0.001
Minimal or Mild	20/47 (42.6)	86/114 (75.4)	
Moderate or Marked	27/47 (57.4)	28/114 (24.5)	
Rim Artifacts			1.000
Absent	42/47 (89.3)	100/114 (87.7)	
Present	5/47 (10.7)	14/114 (12.3)	
Ripple Artifacts			0.005
Absent	36/47 (76.6)	60/114 (52.6)	
Present	11/47 (23.4)	54/114 (47.4)	
Vascular Artifacts			0.042
Absent	41/47 (87.2)	82/114 (71.9)	
Present	6/47 (12.8)	32/114 (28.1)	
Air Trapping Artifacts			0.104
Absent	43/47 (87.2)	92/114 (71.9)	
Present	4/47 (12.8)	22/114 (28.1)	
SNR^a^	183.2 ± 134.0	359.7 ± 141.9	< 0.001
CNR^a^	194.5 ± 153.0	431.7 ± 193.1	< 0.001
BCR^a^	108.6 ± 84.4	230.2 ± 103.2	< 0.001

*BPE* background parenchymal enhancement, *SNR* signal-to-noise ratio, *CNR* contrast-to-noise ratio, *BCR* background contrast ratio

^a^ Data are shown as the mean values ± standard deviations. Other data are shown as proportions with percentages in parentheses

### Performance of Radiomics models based on cross-validation results

For the LASSO regression models, the average AUC, accuracy, sensitivity, and specificity values were 0.926 ± 0.047, 0.895 ± 0.061, 0.891 ± 0.085, and 0.908 ± 0.096, respectively. For the RF models, the average AUC, accuracy, sensitivity, and specificity values were 0.915 ± 0.055, 0.880 ± 0.068, 0.878 ± 0.097, and 0.886 ± 0.108, respectively. The statistics of the features used ≥20% of the times by LASSO and features with the largest permutation importance scores generated by RF in the cross-validation are given in the Supplemental Tables 2 and 3.

### Summary of classification results for the lesions

**Fig. 2:**
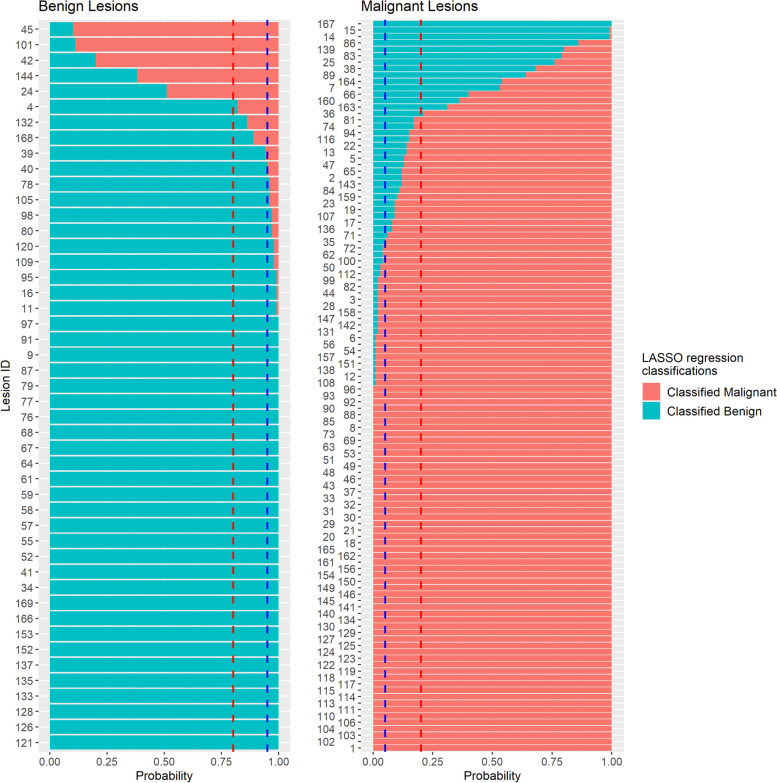
Least absolute shrinkage and selection operator (LASSO) regression radiomics model classification results for 100 rounds of cross-validation. The blue dashed line is the cutoff line for a misclassification probability of 0.05, and the red dashed line is the cutoff line for a misclassification probability of 0.20 for benign and malignant lesions. The average AUC, accuracy, sensitivity, and specificity values and the standard deviation are 0.926 ± 0.047, 0.895 ± 0.061, 0.891 ± 0.085, and 0.908 ± 0.096

**Fig. 3:**
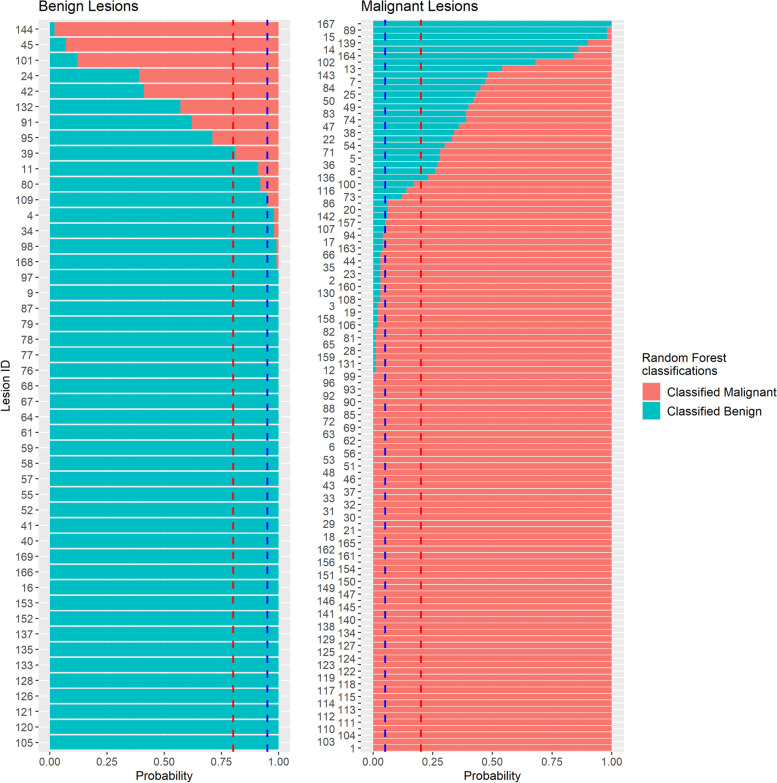
Random forest (RF) radiomics model classification results for 100 rounds of cross-validation. The blue dashed line is the cutoff line for a misclassification probability of 0.05, and the red dashed line is the cutoff line for a misclassification probability of 0.20 for benign and malignant lesions. The average AUC, accuracy, sensitivity, and specificity values and the standard deviation are 0.915 ± 0.055, 0.880 ± 0.068, 0.878 ± 0.097, and 0.886 ± 0.108

The lesion classification results are shown in Fig. 2 (LASSO regression) and Fig. 3 (RF). For the LASSO regression models, 20 (12.4%) of the 161 lesions (5 benign; 15 malignant) were incorrectly classified for no less than 20.0% of the 100 iterations, and 116 (72.0%) of the 161 lesions (37 benign; 79 malignant) were incorrectly classified for no more than 5.0% of the iterations; for the RF models, 33 (20.5%) lesions (8 benign; 25 malignant) were misclassified for no less than 20.0% of 100 iterations, and 116 (72.0%) lesions (35 benign; 81 malignant) were incorrectly classified for no more than 5.0% of the iterations. Based on our definition, a total of 16 (9.9%) lesions (5 benign; 11 malignant) were defined as having a high misclassification probability, and 101 (62.7%) lesions (32 benign; 69 malignant) were defined as having a low misclassification probability.

### Factors identified that may influence the classification performance of Radiomics models

**Fig. 4:**
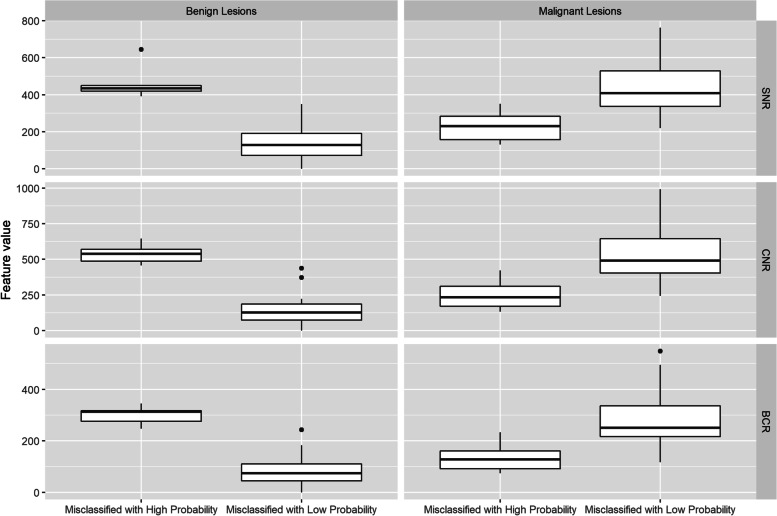
Distribution of values of quantitative features in the subgroups of interest. SNR = signal-to-noise ratio; CNR = contrast-to-noise ratio; BCR = background contrast ratio

A summary of the image features and the objective quantitative features in the subgroups of interest is shown in Table 2. The univariate analysis showed that larger lesion size (*p* = 0.003), the presence of rim artifacts (*p* <  0.001), and ripple artifacts (*p* = 0.042) may increase the misclassification rate for benign lesions. Among the malignant lesions, a smaller lesion size (*p* <  0.001) was found to be a factor that may be associated with misclassification. The distributions of the objective quantitative features are shown in Fig. 4. Among the benign lesions, compared with lesions with a low misclassification probability, lesions with a high misclassification probability showed higher values for the SNR, CNR, and BCR. Among the malignant lesions, compared with the lesions with low misclassification probability, the lesions with high misclassification probability showed lower values for the SNR, CNR, and BCR. All of the differences between the lesions with a high misclassification probability and lesions with a low misclassification probability were statistically significant (*p* <  0.050).

**Table 2:** Summary of image features and objective quantitative features in subgroups of interest

Image features	Category	Benign lesions		*P* value	Malignant lesions		*P* value
		High Misclassification Probability (*n* = 5)	Low Misclassification Probability (*n* = 32)		High Misclassification Probability (*n* = 11)	Low Misclassification Probability (*n* = 69)	
Lesion size ^a^	/	31.3 ± 11.8	11.8 ± 9.2	0.003	17.0 ± 6.0	34.4 ± 16.8	< 0.001
Breast density	a-b	1/5 (40.0)	2/32 (6.3)	0.362	2/11 (18.2)	23/69 (33.3)	0.488
	c-d	4/5 (60.0)	30/32 (93.8)		9/11 (81.8)	46/69 (66.7)	
Degree of BPE	Minimal or mild	1/5 (20.0)	14/32 (43.8)	0.629	10/11 (90.9)	52/69 (75.4)	0.440
	Moderate or marked	4/5 (80.0)	18/32 (56.3)		1/11 (9.1)	17/69 (24.6)	
Rim artifact	Absent	1/5 (20.0)	31/32 (96.9)	< 0.001	10/11 (90.9)	59/69 (85.5)	0.999
	Present	4/5 (80.0)	1/32 (3.1)		1/11 (9.1)	10/69 (14.5)	
Ripple artifact	Absent	3/5 (60.0)	31/32 (96.9)	0.042	6/11 (54.5)	33/69 (47.8)	0.753
	Present	2/5 (40.0)	1/32 (3.1)		5/11 (45.5)	36/69 (52.2)	
Vascular artifact	Absent	4/5 (80.0)	29/32 (90.6)	0.456	9/11 (81.8)	48/69 (69.6)	0.497
	Present	1/5 (20.0)	3/32 (9.4)		2/11 (18.2)	21/69 (30.4)	
Air trapping artifact	Absent	5/5 (100.0)	31/32 (96.9)	0.255	7/11 (63.6)	57/69 (82.6)	0.217
	Present	0/5 (0.0)	1/32 (3.1)		4/11 (36.4)	12/69 (17.4)	
SNR ^a^	/	467.6 ± 100.8	143.9 ± 87.3	< 0.001	224.1 ± 75.9	410.4 ± 130.0	< 0.001
CNR ^a^	/	539.7 ± 73.8	149.5 ± 98.4	< 0.001	247.3 ± 92.1	505.2 ± 179.9	< 0.001
BCR ^a^	/	299.8 ± 38.4	83.8 ± 53.4	< 0.001	133.6 ± 49.7	265.7 ± 93.8	< 0.001

*BPE* background parenchymal enhancement, *SNR* signal-to-noise ratio, *CNR* contrast-to-noise ratio, *BCR* background contrast ratio

^a^ Data are shown as the mean values ± standard deviations. Other data are shown as proportions with percentages in parentheses

Multivariate analysis was only performed in the malignant lesion group since the small number of lesions in the benign lesion group prevented the logistic regression model from converging. In Table 3, the results show that a smaller lesion size (odds ratio [OR] = 0.699, *p* = 0.002) and the presence of air trapping artifacts (OR = 36.568, *p* = 0.025) may be factors that may result in the misclassification of malignant lesions.

**Table 3:** Multivariate factor analysis results for malignant lesions in the subgroups of interest

Image features	OR	95% CI	*P* value
Lesion size ^a^	0.699	(0.528, 0.837)	0.002
Breast density (c-d)	12.619	(1.216, 381.215)	0.068
Degree of BPE (moderate or marked)	1.517	(0.036, 50.007)	0.811
Presence of rim artifacts	0.064	(0.006, 17.652)	0.815
Presence of vascular artifacts	0.442	(0.010, 6.336)	0.594
Presence of ripple artifacts	2.795	(0.400, 24.149)	0.310
Presence of air trapping artifacts	36.568	(2.205, 1665.08)	0.025

*BPE* background parenchymal enhancement, *OR* odds ratio, *CI* confidence interval

^a^ OR (95% CI): The effect size is calculated based on each 1 mm change in the variable

In addition, both the univariate and multivariate analyses based on the LASSO regression models and RF models showed similar results (Supplemental Table 4-Table 7).

### Results of additional exploratory analyses

#### Correct classification rates for lesions with/without influential factors

A summary of correct classification rates between lesions with and without certain influential factor is given in Table 4. A smaller lesion size (< 20 mm) increased the correct classification rate among benign lesions by 0.223 ± 0.098 (mean ± standard deviation) and 0.231 ± 0.095, and decreased the correct classification rate among malignant lesions by − 0.140 ± 0.049 and − 0.256 ± 0.069 for LASSO and RF, respectively. The present of rim artifacts decreased the correct classification rate among benign lesions by − 0.613 ± 0.193 and − 0.624 ± 0.140 for LASSO and RF, respectively. The present of ripple artifacts decreased the correct classification rate among benign lesions by − 0.126 ± 0.075 and − 0.165 ± 0.106 for LASSO and RF, respectively. The present of air trapping artifacts decreased the correct classification rate among malignant lesions by − 0.148 ± 0.056 and − 0.088 ± 0.054 for LASSO and RF, respectively. However, the presence of both smaller lesion size and air trapping artifacts decreased the correct classification rate among malignant lesions by − 0.458 ± 0.168 and − 0.559 ± 0.145 for LASSO and RF, respectively.

**Table 4:**
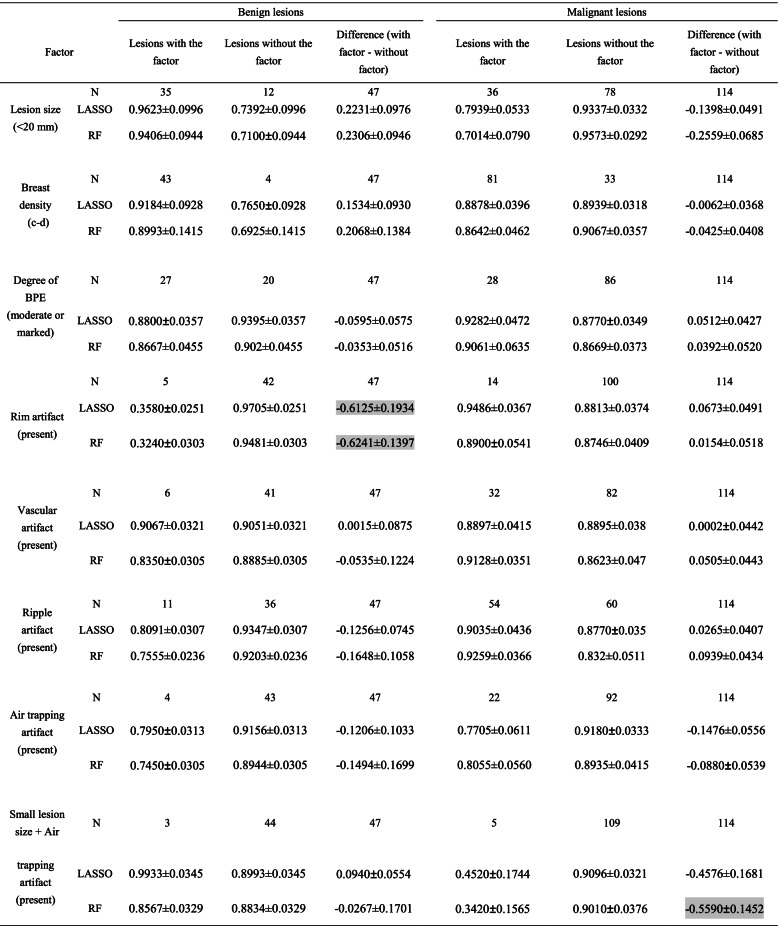
Summary of additional exploratory analysis for correct classification rates between lesions with and without influential factors

The data are presented as the mean correct classification rate ± standard deviation across the 100 rounds of cross-validation. Values with an absolute difference in the correct classification rate equal to or larger than 0.5000 are marked with gray

#### Performance of Radiomics models in the data with/without influential factors

We performed two more sets of 100 rounds of cross-validations among the data on the lesions with or without rim artifacts, ripple artifacts, and/or air trapping artifacts (with: 87 in total, 16 benign and 71 malignant; without: 74 in total, 31 benign and 43 malignant). We only considered valid classification results without prediction issues due to the small number of prediction categories. For the LASSO regression models in lesions with/without the abovementioned artifacts, the average AUC, accuracy, sensitivity, and specificity values were 0.875 ± 0.078 vs. 0.970 ± 0.071, 0.858 ± 0.097 vs. 0.965 ± 0.066, 0.851 ± 0.099 vs. 0.967 ± 0.088, and 0.898 ± 0.123 vs. 0.967 ± 0.092, respectively. For the RF models in lesions with/without the abovementioned artifacts, the average AUC, accuracy, sensitivity, and specificity values were 0.852 ± 0.085 vs. 0.961 ± 0.094, 0.830 ± 0.100 vs. 0.952 ± 0.079, 0.822 ± 0.123 vs. 0.953 ± 0.121, and 0.907 ± 0.124 vs. 0.968 ± 0.090, respectively.

## Discussion

Overall, the performance of the two algorithms (LASSO and RF) used in this study was comparable to that of the models in the published literature using radiomics features of CEM to classify breast lesions (AUC = 0.848–0.950, accuracy = 78.4–90.0%) [13–16].

**Fig. 5:**
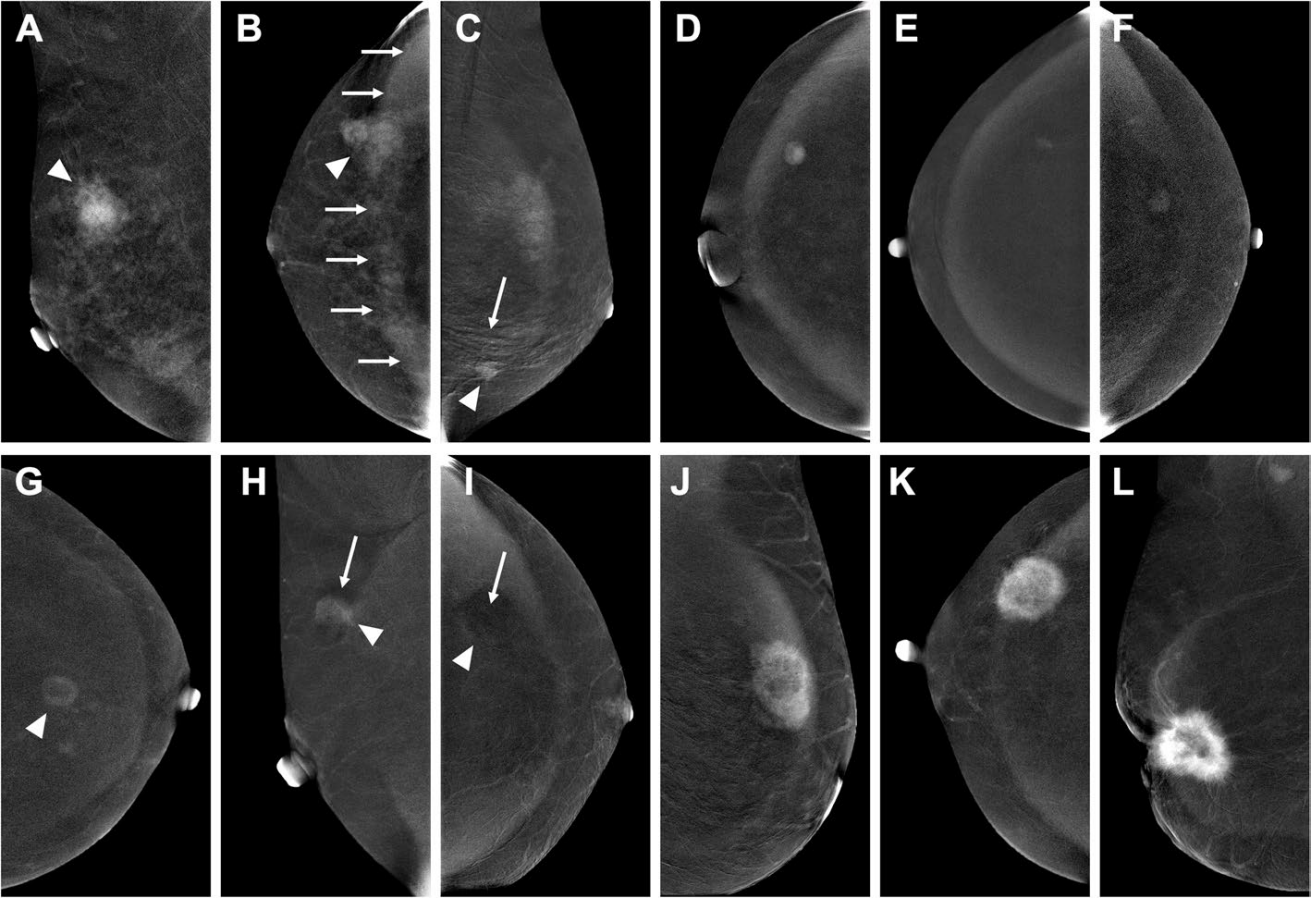
Examples of dual-energy subtraction (DES) images of contrast-enhanced mammography (CEM) classified by the radiomics models. **A**-**C** Examples of benign lesions with high misclassification probabilities. The lesions are annotated with arrowheads. **A** A 42-year-old woman with a markedly enhanced lesion in the upper quadrant of the right breast. Biopsy revealed a fibroadenoma. The diameter of the lesion is 31.5 mm (mean lesion size of all the benign lesions: 17.1 mm). The patient has marked BPE. **B** A 47-year-old woman with a moderately enhanced lesion in the outer quadrant of the right breast. Biopsy revealed adenosis with a fibroadenoma. Rim artifacts are present at the location of the lesion (arrows). The patient has marked BPE. **C** A 35-year-old woman with a moderately enhanced lesion in the lower quadrant of the left breast. Biopsy revealed an intraductal papilloma. Ripple artifacts are present at the location of the lesion (arrow). The patient has mild BPE. **D**-**F** Examples of benign lesions with low misclassification probabilities. **D** A 50-year-old woman with a moderately enhanced lesion in the outer quadrant of the right breast. Biopsy revealed a fibroadenoma. The diameter of the lesion is 10.5 mm. The patient has minimal BPE. **E** A 55-year-old woman with a mildly enhanced lesion in the outer quadrant of the right breast. Biopsy revealed a fibroadenoma. The diameter of the lesion is 8.0 mm. The patient has minimal BPE. **F** A 58-year-old woman with a mildly enhanced lesion in the outer quadrant of the left breast. Biopsy revealed a fibroadenoma. The diameter of the lesion is 10.3 mm. The patient has minimal BPE. **G**-**I** Examples of malignant lesions with high misclassification probabilities. The lesions are annotated with arrowheads. **G** A 60-year-old woman with a mildly enhanced lesion in the central area of the left breast. Biopsy revealed IDC with mucous secretion (grade III). The diameter of the lesion is 16.0 mm (mean lesion size of all malignant lesions: 28.8 mm). The patient has minimal BPE. **H** A 53-year-old woman with a moderately enhanced lesion in the upper quadrant of the right breast. Biopsy revealed IDC (grade II). The diameter of the lesion is 16.3 mm. The patient has minimal BPE with an air trapping artifact in the lesion area (arrow). **I** A 57-year-old woman with a lesion showing negative enhancement in the outer quadrant of the left breast. Biopsy revealed mucous adenocarcinoma. The diameter of the lesion is 27.5 mm. The patient has minimal BPE with negative enhancement artifacts (eclipse sign) in the lesion area (arrow). **J**-**L** Examples of malignant lesions with low misclassification probabilities. **J** A 58-year-old woman with a markedly enhanced lesion in the upper quadrant of the left breast. Biopsy revealed IDC (grade II). The diameter of the lesion is 31.0 mm. The patient has mild BPE. **K** A 49-year-old woman with a markedly enhanced lesion in the outer quadrant of the right breast. Biopsy revealed IDC (grade II). The diameter of the lesion is 39.5 mm. The patient has minimal BPE. **L** A 60-year-old woman with a markedly enhanced lesion in the retro-areola region of the right breast. Biopsy revealed IDC (grade II). The diameter of the lesion is 48.8 mm. The patient has minimal BPE. BPE = background parenchymal enhancement; IDC = invasive ductal carcinoma

The results of factor analyses showed that small lesion size and the presence of rim artifacts, ripple artifacts, and air trapping artifacts might influence classification performances in the LASSO regression models and RF radiomics models. To illustrate the findings, we provided a set of CEM images as examples in Fig. 5. As shown in Fig. 5A-C, benign lesions with larger lesion size and presenting with rim artifacts or ripple artifacts were more likely to be misclassified. Benign lesions that were less likely to be misclassified (Fig. 5D-F) were smaller in size and generally did not contain rim or ripple artifacts. In Fig. 5G-H, malignant lesions with smaller lesion size and presenting with air trapping artifacts were more likely to be misclassified. Malignant lesions that were less likely to be misclassified were generally larger and did not present with air trapping artifacts (Fig. 5J-L). The presence of artifacts seemed to be an influential factor that resulted in misclassification, and the influence could be bidirectional: some artifacts, such as rim artifacts and ripple artifacts, tended to influence the classification of a lesion as malignant, probably because these artifacts increase the signal intensity and/or heterogeneity of the lesions, while other artifacts, such as air trapping artifacts and negative enhancement artifacts, decrease the signal intensity of the lesions. Thus, lesions with such artifacts might be more likely to be classified as benign.

Our findings were further examined by the results of additional exploratory analyses. Based on the cross-validation results, correct classification rates could obviously decrease (approximately 50% on average) for benign lesions with rim artifacts and smaller malignant lesions (< 20 mm) with air trapping artifacts. Furthermore, model accuracy could obviously decrease by an average of 10–12% when the analyses were only performed for lesions with rim artifacts, ripple artifacts, and/or air trapping artifacts versus lesions without the artifacts.

Our findings could also be potentially explained by objective quantitative image features in an interpretable way. The SNR, CNR, and BCR values showed significantly different distributions between lesions with high misclassification probability and lesions with low misclassification probability in both the benign and malignant lesion groups. These results were also in line with the abovementioned findings and inferences. It is worth mentioning that the quantitative features may be associated with the presence of artifacts as well, so we did not include these features in our exploratory analyses. Benign lesions with high misclassification probability showed higher signal intensity after enhancement (Fig. 5A-C), while malignant lesions with high misclassification probability showed lower signal intensity (Fig. 5G-I). Several aspects could contribute to high lesion signal intensity, including the inherent characteristics of the lesion itself and external influential factors, which may further cause lesion misclassification by the radiomics models. Several quantitative studies of CEM have demonstrated that malignant lesions tend to show more obvious enhancement than benign lesions [33–35]. Some studies [36, 37] have noted that the enhancement intensity depends on the size of the tumor and is more obvious for larger lesions than for smaller lesions. In other words, larger benign lesions can also display strong enhancement, and smaller malignant lesions can also display slight enhancement. Furthermore, as reported by Yagil et al. [38], rim and ripple artifacts were the main artifacts commonly seen on CEM. Researchers [23, 39, 40] have stated that DES images are prone to rim artifacts of increased density as a result of radiation scattering. Additionally, BPE, which refers to the uptake of contrast medium by normal fibroglandular breast tissue [41, 42], may also add the signal intensity of the lesions. In contrast, air trapping artifacts, which represent the presence of air and create a dark area due to incomplete contact between the skin and the detector or compression paddle [23, 24], may result in more neutral signal intensity.

Although some scholars have considered that some artifacts in CEM images might not compromise image quality [24, 38], we found that some artifacts in CEM images might affect the diagnostic performance of radiomics models, and other scholars [23, 43, 44] have proposed that some artifacts may present challenges to image interpretation. Therefore, it is still necessary to stress the importance of high-quality images. Neppalli et al. reported [45] that the type, incidence, and severity of CEM-specific artifacts differ between image device vendors. To date, several image-processing algorithms have been developed to reduce artifacts and improve the image quality [46–48]. For example, scatter correction techniques are becoming commercially available [48], and the rim artifacts are not present in the newer systems [24]. Furthermore, except for equipment- or technique-related factors, CEM-specific artifacts can also be alleviated by patient- or technologist-related factors. Therefore, it is also important to use standard and appropriate protocols during image acquisition and perform regular quality control tests [49] to prevent or minimize these artifacts.

There are some limitations in our study. First, the relatively small sample size is the main limitation. A larger sample may help provide more information with the same accuracy. Second, radiomics features derived from CEM, in general, could have inherent limitations caused by the two-dimensional nature of the images and compression. Third, more homogeneous baseline characteristics between benign and malignant lesions may potentially help better interpret the results. To avoid bias, we used 100 rounds of cross-validation instead of a single round to obtain “averaged” classification results. Performing factor analysis separately for benign and malignant lesions could further limit the impact of unbalanced characteristics.

## Conclusions

Our study found that large lesion size and the presence of rim and/or ripple artifacts were associated with misclassification of benign lesions, and small lesion size and presence of air trapping artifacts were associated with misclassification of malignant lesions. The results imply that we should be aware that the results of radiomics models could be less reliable when these influential factors are present. Based on these findings, some methods, such as alleviating artifacts by using specific postprocessing algorithms [48], applying adequate compression of the breast [24], referring to the image information around the lesion [50], and employing an adjusted algorithm that considers these influential factors, can potentially help to build more accurate and interpretable radiomics classification models.

## Supplementary Information

**Additional file 1.**

**Additional file 2.**

## Data Availability

The datasets generated and/or analysed during the current study are not publicly available due confidential information but are available from the corresponding author on reasonable request.

## Abbreviations

AUC: area under the curve
BPE: background parenchymal enhancement
CC: craniocaudal
CEM: contrast-enhanced mammography
CI: confidence interval
DES: dual-energy subtraction
GLCM: gray-level cooccurrence matrix
GLRLM: gray-level run length matrix
GLSZM: gray-level size zone matrix
HE: high-energy
LASSO: least absolute shrinkage and selection operator
LE: low-energy
MLO: mediolateral oblique
OR: odds ratio
RF: random forest
