# Supplementary material for: Identifying factors that may influence the classification performance of radiomics models using contrast-enhanced mammography (CEM) images
Source: Cancer Imaging. 2022 May 12;22:22. doi: 10.1186/s40644-022-00460-8 (PMC9101829; doi:10.1186/s40644-022-00460-8)
Supplement: Supplementary file 1 — Additional file 1. [file 40644_2022_460_MOESM1_ESM.docx]

**Supplemental Materials**

**E1. Process and Calculating Method of the Objective Quantitative Features**

For the objective quantitative assessment, a four-step method was employed in this study.

First, a circular region-of-interest (ROI) of about 1 cm^2^ [1] was manually placed over the most evident enhancement area within the lesion. If the lesion is not large enough to contain this area of interest, the placed ROI should be as large as possible.

Second, another ROI of 1 cm^2^ was put in the most homogenous background area, avoiding the lesion, pectoral muscle and artifact areas.

Third, the mean density and standard deviation (SD) values of the lesion ROI and background ROI were recorded, respectively.

Forth, signal-to-noise ratio (SNR), contrast-to-noise ratio (CNR) and background contrast ratio (BCR) were calculated for each lesion using the following formulas [2-4]:

$SNR=\frac{{SI}_{l}-{SI}_{b}}{\sigma_{l}}\times100\times100\%$ (1)

$CNR=\frac{{SI}_{l}-{SI}_{b}}{\sigma_{b}}\times100\times100\%$ (2)

$BCR=\frac{{SI}_{l}-{SI}_{b}}{{SI}_{l}+{SI}_{b}}\times10000$ (3)

Where ${SI}_{l}$ represents mean density value of the lesion ROI, ${SI}_{b}$ represents mean density value of the background ROI, $\sigma_{l}$ represents SD value of the lesion ROI, and $\sigma_{b}$ represents SD value of the background ROI.

**E2. Statistical Details of 100 Rounds Cross Validation and Obtaining Misclassification Probability**

To obtain the misclassification probability for each lesion and evaluate the performance of the two methods, we conducted the following iterative procedure:

Step 1. Randomly split the data into five parts.

Step 2. We used four of the five parts as a training dataset (80% of the whole data) to train a LASSO regression model and an RF model. The remaining part was used as a testing dataset. Adjusted weights inversely proportion to frequencies of malignant and benign lesions were calculated based on the training dataset and incorporated in the model building for both algorithms. In the training dataset, we performed the normalization using training dataset and applied the mean and standard deviation obtained from the training dataset to the testing dataset to get the normalized features accordingly. Then, we removed the highly correlated redundant radiomics features if the pairwise correlations were greater than 0.8. Specifically, if two radiomics features had a correlation greater than 0.8, the radiomics feature with the largest mean absolute correlation was removed. The number of trees used in the RF model was set to 1000. Then, both models were used to classify the lesions as benign or malignant in the testing dataset. The classification results of the lesions in the testing dataset were obtained.

Step 3. We repeated step 2 to let each part of the data be predicted by the classification models built based on the other four parts. Then, we obtained a LASSO regression classification result and a RF classification result for each lesion. Every time a new LASSO regression model and a new RF model were built, the area under the curve (AUC), accuracy, sensitivity, and specificity values in the testing dataset were calculated.

Step 4. We repeated step 1 to step 3 for one hundred iterations, which is equivalent to building five hundred LASSO regression models and five hundred RF models. After the procedure, for each lesion, we obtained one hundred classification results by the LASSO regression models and RF models. Then, the misclassification probability of each lesion was calculated by the number of times the lesion was incorrectly classified divided by one hundred. The overall performance of each algorithm was evaluated by calculating the mean AUC, accuracy, sensitivity, and specificity values.

**References**

1. Baldelli, P., N. Phelan, and G. Egan, *A novel method for contrast-to-noise ratio (CNR) evaluation of digital mammography detectors.* Eur Radiol, 2009. **19**(9): p. 2275-85.

2. Tamura, T., et al., *How to Improve the Conspicuity of Breast Tumors on Computed High b-value Diffusion-weighted Imaging.* Magn Reson Med Sci, 2019. **18**(2): p. 119-125.

3. Rudnicki, W., et al., *Correlation between quantitative assessment of contrast enhancement in contrast-enhanced spectral mammography (CESM) and histopathology-preliminary results.* Eur Radiol, 2019. **29**(11): p. 6220-6226.

4. Price, R.R., et al., *Quality assurance methods and phantoms for magnetic resonance imaging: Report of AAPM nuclear magnetic resonance Task Group No. 1.* Medical Physics, 1990. **17**(2): p. 287-295.
