# Supplementary material for: Identifying factors that may influence the classification performance of radiomics models using contrast-enhanced mammography (CEM) images
Source: Cancer Imaging. 2022 May 12;22:22. doi: 10.1186/s40644-022-00460-8 (PMC9101829; doi:10.1186/s40644-022-00460-8)
Supplement: Supplementary file 2 — Additional file 2. [file 40644_2022_460_MOESM2_ESM.docx]

**Supplemental Tables**

Supplemental Table 1. All of the extracted radiomic features in the study.

| Feature types | Number | Radiomics features |
| --- | --- | --- |
| Shape features | 1 | original_shape_SurfaceVolumeRatio |
|  | 2 | original_shape_MeshVolume |
|  | 3 | original_shape_Elongation |
|  | 4 | original_shape_MajorAxisLength |
|  | 5 | original_shape_SurfaceArea |
|  | 6 | original_shape_Sphericity |
|  | 7 | original_shape_MinorAxisLength |
|  | 8 | original_shape_Maximum2DDiameterRow |
|  | 9 | original_shape_Flatness |
|  | 10 | original_shape_Maximum3DDiameter |
|  | 11 | original_shape_Maximum2DDiameterColumn |
|  | 12 | original_shape_Maximum2DDiameterSlice |
|  | 13 | original_shape_VoxelVolume |
|  | 14 | original_shape_LeastAxisLength |
| First order features | 1 | original_firstorder_Kurtosis |
|  | 2 | original_firstorder_TotalEnergy |
|  | 3 | original_firstorder_Skewness |
|  | 4 | original_firstorder_Median |
|  | 5 | original_firstorder_InterquartileRange |
|  | 6 | original_firstorder_10Percentile |
|  | 7 | original_firstorder_RobustMeanAbsoluteDeviation |
|  | 8 | original_firstorder_90Percentile |
|  | 9 | original_firstorder_MeanAbsoluteDeviation |
|  | 10 | original_firstorder_Variance |
|  | 11 | original_firstorder_Uniformity |
|  | 12 | original_firstorder_Maximum |
|  | 13 | original_firstorder_Minimum |
|  | 14 | original_firstorder_Mean |
|  | 15 | original_firstorder_RootMeanSquared |
|  | 16 | original_firstorder_Range |
|  | 17 | original_firstorder_Entropy |
|  | 18 | original_firstorder_Energy |
| GCLM features | 1 | original_glcm_MCC |
|  | 2 | original_glcm_MaximumProbability |
|  | 3 | original_glcm_Correlation |
|  | 4 | original_glcm_Autocorrelation |
|  | 5 | original_glcm_Id |
|  | 6 | original_glcm_ClusterShade |
|  | 7 | original_glcm_JointAverage |
|  | 8 | original_glcm_Idm |
|  | 9 | original_glcm_SumSquares |
|  | 10 | original_glcm_SumEntropy |
|  | 11 | original_glcm_Idmn |
|  | 12 | original_glcm_JointEntropy |
|  | 13 | original_glcm_DifferenceVariance |
|  | 14 | original_glcm_Imc2 |
|  | 15 | original_glcm_DifferenceEntropy |
|  | 16 | original_glcm_DifferenceAverage |
|  | 17 | original_glcm_Idn |
|  | 18 | original_glcm_JointEnergy |
|  | 19 | original_glcm_ClusterProminence |
|  | 20 | original_glcm_InverseVariance |
|  | 21 | original_glcm_ClusterTendency |
|  | 22 | original_glcm_SumAverage |
|  | 23 | original_glcm_Imc1 |
|  | 24 | original_glcm_Contrast |
| GLRLM feature | 1 | original_glrlm_GrayLevelVariance |
|  | 2 | original_glrlm_LongRunLowGrayLevelEmphasis |
|  | 3 | original_glrlm_RunPercentage |
|  | 4 | original_glrlm_HighGrayLevelRunEmphasis |
|  | 5 | original_glrlm_GrayLevelNonUniformity |
|  | 6 | original_glrlm_ShortRunEmphasis |
|  | 7 | original_glrlm_LowGrayLevelRunEmphasis |
|  | 8 | original_glrlm_RunLengthNonUniformity |
|  | 9 | original_glrlm_RunVariance |
|  | 10 | original_glrlm_ShortRunLowGrayLevelEmphasis |
|  | 11 | original_glrlm_LongRunEmphasis |
|  | 12 | original_glrlm_ShortRunHighGrayLevelEmphasis |
|  | 13 | original_glrlm_GrayLevelNonUniformityNormalized |
|  | 14 | original_glrlm_LongRunHighGrayLevelEmphasis |
|  | 15 | original_glrlm_RunLengthNonUniformityNormalized |
|  | 16 | original_glrlm_RunEntropy |
| GCSZM features | 1 | original_glszm_ZoneVariance |
|  | 2 | original_glszm_GrayLevelVariance |
|  | 3 | original_glszm_ZoneEntropy |
|  | 4 | original_glszm_SizeZoneNonUniformity |
|  | 5 | original_glszm_GrayLevelNonUniformity |
|  | 6 | original_glszm_LargeAreaHighGrayLevelEmphasis |
|  | 7 | original_glszm_ZonePercentage |
|  | 8 | original_glszm_LargeAreaLowGrayLevelEmphasis |
|  | 9 | original_glszm_SmallAreaHighGrayLevelEmphasis |
|  | 10 | original_glszm_HighGrayLevelZoneEmphasis |
|  | 11 | original_glszm_GrayLevelNonUniformityNormalized |
|  | 12 | original_glszm_LowGrayLevelZoneEmphasis |
|  | 13 | original_glszm_SizeZoneNonUniformityNormalized |
|  | 14 | original_glszm_SmallAreaLowGrayLevelEmphasis |
|  | 15 | original_glszm_LargeAreaEmphasis |
|  | 16 | original_glszm_SmallAreaEmphasis |
| Wavelet features | 1 | wavelet-HLL_glcm_MCC |
|  | 2 | wavelet-HLL_glcm_MaximumProbability |
|  | 3 | wavelet-HLL_glcm_Correlation |
|  | 4 | wavelet-HLL_glcm_Autocorrelation |
|  | 5 | wavelet-HLL_glcm_Id |
|  | 6 | wavelet-HLL_glcm_ClusterShade |
|  | 7 | wavelet-HLL_glcm_JointAverage |
|  | 8 | wavelet-HLL_glcm_Idm |
|  | 9 | wavelet-HLL_glcm_SumSquares |
|  | 10 | wavelet-HLL_glcm_SumEntropy |
|  | 11 | wavelet-HLL_glcm_Idmn |
|  | 12 | wavelet-HLL_glcm_JointEntropy |
|  | 13 | wavelet-HLL_glcm_DifferenceVariance |
|  | 14 | wavelet-HLL_glcm_Imc2 |
|  | 15 | wavelet-HLL_glcm_DifferenceEntropy |
|  | 16 | wavelet-HLL_glcm_DifferenceAverage |
|  | 17 | wavelet-HLL_glcm_Idn |
|  | 18 | wavelet-HLL_glcm_JointEnergy |
|  | 19 | wavelet-HLL_glcm_ClusterProminence |
|  | 20 | wavelet-HLL_glcm_InverseVariance |
|  | 21 | wavelet-HLL_glcm_ClusterTendency |
|  | 22 | wavelet-HLL_glcm_SumAverage |
|  | 23 | wavelet-HLL_glcm_Imc1 |
|  | 24 | wavelet-HLL_glcm_Contrast |
|  | 25 | wavelet-HLL_glszm_ZoneVariance |
|  | 26 | wavelet-HLL_glszm_GrayLevelVariance |
|  | 27 | wavelet-HLL_glszm_ZoneEntropy |
|  | 28 | wavelet-HLL_glszm_SizeZoneNonUniformity |
|  | 29 | wavelet-HLL_glszm_GrayLevelNonUniformity |
|  | 30 | wavelet-HLL_glszm_LargeAreaHighGrayLevelEmphasis |
|  | 31 | wavelet-HLL_glszm_ZonePercentage |
|  | 32 | wavelet-HLL_glszm_LargeAreaLowGrayLevelEmphasis |
|  | 33 | wavelet-HLL_glszm_SmallAreaHighGrayLevelEmphasis |
|  | 34 | wavelet-HLL_glszm_HighGrayLevelZoneEmphasis |
|  | 35 | wavelet-HLL_glszm_GrayLevelNonUniformityNormalized |
|  | 36 | wavelet-HLL_glszm_LowGrayLevelZoneEmphasis |
|  | 37 | wavelet-HLL_glszm_SizeZoneNonUniformityNormalized |
|  | 38 | wavelet-HLL_glszm_SmallAreaLowGrayLevelEmphasis |
|  | 39 | wavelet-HLL_glszm_LargeAreaEmphasis |
|  | 40 | wavelet-HLL_glszm_SmallAreaEmphasis |
|  | 41 | wavelet-HLL_firstorder_Kurtosis |
|  | 42 | wavelet-HLL_firstorder_TotalEnergy |
|  | 43 | wavelet-HLL_firstorder_Skewness |
|  | 44 | wavelet-HLL_firstorder_Median |
|  | 45 | wavelet-HLL_firstorder_InterquartileRange |
|  | 46 | wavelet-HLL_firstorder_10Percentile |
|  | 47 | wavelet-HLL_firstorder_RobustMeanAbsoluteDeviation |
|  | 48 | wavelet-HLL_firstorder_90Percentile |
|  | 49 | wavelet-HLL_firstorder_MeanAbsoluteDeviation |
|  | 50 | wavelet-HLL_firstorder_Variance |
|  | 51 | wavelet-HLL_firstorder_Uniformity |
|  | 52 | wavelet-HLL_firstorder_Maximum |
|  | 53 | wavelet-HLL_firstorder_Minimum |
|  | 54 | wavelet-HLL_firstorder_Mean |
|  | 55 | wavelet-HLL_firstorder_RootMeanSquared |
|  | 56 | wavelet-HLL_firstorder_Range |
|  | 57 | wavelet-HLL_firstorder_Entropy |
|  | 58 | wavelet-HLL_firstorder_Energy |
|  | 59 | wavelet-HLL_glrlm_GrayLevelVariance |
|  | 60 | wavelet-HLL_glrlm_LongRunLowGrayLevelEmphasis |
|  | 61 | wavelet-HLL_glrlm_RunPercentage |
|  | 62 | wavelet-HLL_glrlm_HighGrayLevelRunEmphasis |
|  | 63 | wavelet-HLL_glrlm_GrayLevelNonUniformity |
|  | 64 | wavelet-HLL_glrlm_ShortRunEmphasis |
|  | 65 | wavelet-HLL_glrlm_LowGrayLevelRunEmphasis |
|  | 66 | wavelet-HLL_glrlm_RunLengthNonUniformity |
|  | 67 | wavelet-HLL_glrlm_RunVariance |
|  | 68 | wavelet-HLL_glrlm_ShortRunLowGrayLevelEmphasis |
|  | 69 | wavelet-HLL_glrlm_LongRunEmphasis |
|  | 70 | wavelet-HLL_glrlm_ShortRunHighGrayLevelEmphasis |
|  | 71 | wavelet-HLL_glrlm_GrayLevelNonUniformityNormalized |
|  | 72 | wavelet-HLL_glrlm_LongRunHighGrayLevelEmphasis |
|  | 73 | wavelet-HLL_glrlm_RunLengthNonUniformityNormalized |
|  | 74 | wavelet-HLL_glrlm_RunEntropy |
|  | 75 | wavelet-LLH_glcm_MCC |
|  | 76 | wavelet-LLH_glcm_MaximumProbability |
|  | 77 | wavelet-LLH_glcm_Correlation |
|  | 78 | wavelet-LLH_glcm_Autocorrelation |
|  | 79 | wavelet-LLH_glcm_Id |
|  | 80 | wavelet-LLH_glcm_ClusterShade |
|  | 81 | wavelet-LLH_glcm_JointAverage |
|  | 82 | wavelet-LLH_glcm_Idm |
|  | 83 | wavelet-LLH_glcm_SumSquares |
|  | 84 | wavelet-LLH_glcm_SumEntropy |
|  | 85 | wavelet-LLH_glcm_Idmn |
|  | 86 | wavelet-LLH_glcm_JointEntropy |
|  | 87 | wavelet-LLH_glcm_DifferenceVariance |
|  | 88 | wavelet-LLH_glcm_Imc2 |
|  | 89 | wavelet-LLH_glcm_DifferenceEntropy |
|  | 90 | wavelet-LLH_glcm_DifferenceAverage |
|  | 91 | wavelet-LLH_glcm_Idn |
|  | 92 | wavelet-LLH_glcm_JointEnergy |
|  | 93 | wavelet-LLH_glcm_ClusterProminence |
|  | 94 | wavelet-LLH_glcm_InverseVariance |
|  | 95 | wavelet-LLH_glcm_ClusterTendency |
|  | 96 | wavelet-LLH_glcm_SumAverage |
|  | 97 | wavelet-LLH_glcm_Imc1 |
|  | 98 | wavelet-LLH_glcm_Contrast |
|  | 99 | wavelet-LLH_glszm_ZoneVariance |
|  | 100 | wavelet-LLH_glszm_GrayLevelVariance |
|  | 101 | wavelet-LLH_glszm_ZoneEntropy |
|  | 102 | wavelet-LLH_glszm_SizeZoneNonUniformity |
|  | 103 | wavelet-LLH_glszm_GrayLevelNonUniformity |
|  | 104 | wavelet-LLH_glszm_LargeAreaHighGrayLevelEmphasis |
|  | 105 | wavelet-LLH_glszm_ZonePercentage |
|  | 106 | wavelet-LLH_glszm_LargeAreaLowGrayLevelEmphasis |
|  | 107 | wavelet-LLH_glszm_SmallAreaHighGrayLevelEmphasis |
|  | 108 | wavelet-LLH_glszm_HighGrayLevelZoneEmphasis |
|  | 109 | wavelet-LLH_glszm_GrayLevelNonUniformityNormalized |
|  | 110 | wavelet-LLH_glszm_LowGrayLevelZoneEmphasis |
|  | 111 | wavelet-LLH_glszm_SizeZoneNonUniformityNormalized |
|  | 112 | wavelet-LLH_glszm_SmallAreaLowGrayLevelEmphasis |
|  | 113 | wavelet-LLH_glszm_LargeAreaEmphasis |
|  | 114 | wavelet-LLH_glszm_SmallAreaEmphasis |
|  | 115 | wavelet-LLH_firstorder_Kurtosis |
|  | 116 | wavelet-LLH_firstorder_TotalEnergy |
|  | 117 | wavelet-LLH_firstorder_Skewness |
|  | 118 | wavelet-LLH_firstorder_Median |
|  | 119 | wavelet-LLH_firstorder_InterquartileRange |
|  | 120 | wavelet-LLH_firstorder_10Percentile |
|  | 121 | wavelet-LLH_firstorder_RobustMeanAbsoluteDeviation |
|  | 122 | wavelet-LLH_firstorder_90Percentile |
|  | 123 | wavelet-LLH_firstorder_MeanAbsoluteDeviation |
|  | 124 | wavelet-LLH_firstorder_Variance |
|  | 125 | wavelet-LLH_firstorder_Uniformity |
|  | 126 | wavelet-LLH_firstorder_Maximum |
|  | 127 | wavelet-LLH_firstorder_Minimum |
|  | 128 | wavelet-LLH_firstorder_Mean |
|  | 129 | wavelet-LLH_firstorder_RootMeanSquared |
|  | 130 | wavelet-LLH_firstorder_Range |
|  | 131 | wavelet-LLH_firstorder_Entropy |
|  | 132 | wavelet-LLH_firstorder_Energy |
|  | 133 | wavelet-LLH_glrlm_GrayLevelVariance |
|  | 134 | wavelet-LLH_glrlm_LongRunLowGrayLevelEmphasis |
|  | 135 | wavelet-LLH_glrlm_RunPercentage |
|  | 136 | wavelet-LLH_glrlm_HighGrayLevelRunEmphasis |
|  | 137 | wavelet-LLH_glrlm_GrayLevelNonUniformity |
|  | 138 | wavelet-LLH_glrlm_ShortRunEmphasis |
|  | 139 | wavelet-LLH_glrlm_LowGrayLevelRunEmphasis |
|  | 140 | wavelet-LLH_glrlm_RunLengthNonUniformity |
|  | 141 | wavelet-LLH_glrlm_RunVariance |
|  | 142 | wavelet-LLH_glrlm_ShortRunLowGrayLevelEmphasis |
|  | 143 | wavelet-LLH_glrlm_LongRunEmphasis |
|  | 144 | wavelet-LLH_glrlm_ShortRunHighGrayLevelEmphasis |
|  | 145 | wavelet-LLH_glrlm_GrayLevelNonUniformityNormalized |
|  | 146 | wavelet-LLH_glrlm_LongRunHighGrayLevelEmphasis |
|  | 147 | wavelet-LLH_glrlm_RunLengthNonUniformityNormalized |
|  | 148 | wavelet-LLH_glrlm_RunEntropy |
|  | 149 | wavelet-HHH_glcm_MCC |
|  | 150 | wavelet-HHH_glcm_MaximumProbability |
|  | 151 | wavelet-HHH_glcm_Correlation |
|  | 152 | wavelet-HHH_glcm_Autocorrelation |
|  | 153 | wavelet-HHH_glcm_Id |
|  | 154 | wavelet-HHH_glcm_ClusterShade |
|  | 155 | wavelet-HHH_glcm_JointAverage |
|  | 156 | wavelet-HHH_glcm_Idm |
|  | 157 | wavelet-HHH_glcm_SumSquares |
|  | 158 | wavelet-HHH_glcm_SumEntropy |
|  | 159 | wavelet-HHH_glcm_Idmn |
|  | 160 | wavelet-HHH_glcm_JointEntropy |
|  | 161 | wavelet-HHH_glcm_DifferenceVariance |
|  | 162 | wavelet-HHH_glcm_Imc2 |
|  | 163 | wavelet-HHH_glcm_DifferenceEntropy |
|  | 164 | wavelet-HHH_glcm_DifferenceAverage |
|  | 165 | wavelet-HHH_glcm_Idn |
|  | 166 | wavelet-HHH_glcm_JointEnergy |
|  | 167 | wavelet-HHH_glcm_ClusterProminence |
|  | 168 | wavelet-HHH_glcm_InverseVariance |
|  | 169 | wavelet-HHH_glcm_ClusterTendency |
|  | 170 | wavelet-HHH_glcm_SumAverage |
|  | 171 | wavelet-HHH_glcm_Imc1 |
|  | 172 | wavelet-HHH_glcm_Contrast |
|  | 173 | wavelet-HHH_glszm_ZoneVariance |
|  | 174 | wavelet-HHH_glszm_GrayLevelVariance |
|  | 175 | wavelet-HHH_glszm_ZoneEntropy |
|  | 176 | wavelet-HHH_glszm_SizeZoneNonUniformity |
|  | 177 | wavelet-HHH_glszm_GrayLevelNonUniformity |
|  | 178 | wavelet-HHH_glszm_LargeAreaHighGrayLevelEmphasis |
|  | 179 | wavelet-HHH_glszm_ZonePercentage |
|  | 180 | wavelet-HHH_glszm_LargeAreaLowGrayLevelEmphasis |
|  | 181 | wavelet-HHH_glszm_SmallAreaHighGrayLevelEmphasis |
|  | 182 | wavelet-HHH_glszm_HighGrayLevelZoneEmphasis |
|  | 183 | wavelet-HHH_glszm_GrayLevelNonUniformityNormalized |
|  | 184 | wavelet-HHH_glszm_LowGrayLevelZoneEmphasis |
|  | 185 | wavelet-HHH_glszm_SizeZoneNonUniformityNormalized |
|  | 186 | wavelet-HHH_glszm_SmallAreaLowGrayLevelEmphasis |
|  | 187 | wavelet-HHH_glszm_LargeAreaEmphasis |
|  | 188 | wavelet-HHH_glszm_SmallAreaEmphasis |
|  | 189 | wavelet-HHH_firstorder_Kurtosis |
|  | 190 | wavelet-HHH_firstorder_TotalEnergy |
|  | 191 | wavelet-HHH_firstorder_Skewness |
|  | 192 | wavelet-HHH_firstorder_Median |
|  | 193 | wavelet-HHH_firstorder_InterquartileRange |
|  | 194 | wavelet-HHH_firstorder_10Percentile |
|  | 195 | wavelet-HHH_firstorder_RobustMeanAbsoluteDeviation |
|  | 196 | wavelet-HHH_firstorder_90Percentile |
|  | 197 | wavelet-HHH_firstorder_MeanAbsoluteDeviation |
|  | 198 | wavelet-HHH_firstorder_Variance |
|  | 199 | wavelet-HHH_firstorder_Uniformity |
|  | 200 | wavelet-HHH_firstorder_Maximum |
|  | 201 | wavelet-HHH_firstorder_Minimum |
|  | 202 | wavelet-HHH_firstorder_Mean |
|  | 203 | wavelet-HHH_firstorder_RootMeanSquared |
|  | 204 | wavelet-HHH_firstorder_Range |
|  | 205 | wavelet-HHH_firstorder_Entropy |
|  | 206 | wavelet-HHH_firstorder_Energy |
|  | 207 | wavelet-HHH_glrlm_GrayLevelVariance |
|  | 208 | wavelet-HHH_glrlm_LongRunLowGrayLevelEmphasis |
|  | 209 | wavelet-HHH_glrlm_RunPercentage |
|  | 210 | wavelet-HHH_glrlm_HighGrayLevelRunEmphasis |
|  | 211 | wavelet-HHH_glrlm_GrayLevelNonUniformity |
|  | 212 | wavelet-HHH_glrlm_ShortRunEmphasis |
|  | 213 | wavelet-HHH_glrlm_LowGrayLevelRunEmphasis |
|  | 214 | wavelet-HHH_glrlm_RunLengthNonUniformity |
|  | 215 | wavelet-HHH_glrlm_RunVariance |
|  | 216 | wavelet-HHH_glrlm_ShortRunLowGrayLevelEmphasis |
|  | 217 | wavelet-HHH_glrlm_LongRunEmphasis |
|  | 218 | wavelet-HHH_glrlm_ShortRunHighGrayLevelEmphasis |
|  | 219 | wavelet-HHH_glrlm_GrayLevelNonUniformityNormalized |
|  | 220 | wavelet-HHH_glrlm_LongRunHighGrayLevelEmphasis |
|  | 221 | wavelet-HHH_glrlm_RunLengthNonUniformityNormalized |
|  | 222 | wavelet-HHH_glrlm_RunEntropy |
|  | 223 | wavelet-LHH_glcm_MCC |
|  | 224 | wavelet-LHH_glcm_MaximumProbability |
|  | 225 | wavelet-LHH_glcm_Correlation |
|  | 226 | wavelet-LHH_glcm_Autocorrelation |
|  | 227 | wavelet-LHH_glcm_Id |
|  | 228 | wavelet-LHH_glcm_ClusterShade |
|  | 229 | wavelet-LHH_glcm_JointAverage |
|  | 230 | wavelet-LHH_glcm_Idm |
|  | 231 | wavelet-LHH_glcm_SumSquares |
|  | 232 | wavelet-LHH_glcm_SumEntropy |
|  | 233 | wavelet-LHH_glcm_Idmn |
|  | 234 | wavelet-LHH_glcm_JointEntropy |
|  | 235 | wavelet-LHH_glcm_DifferenceVariance |
|  | 236 | wavelet-LHH_glcm_Imc2 |
|  | 237 | wavelet-LHH_glcm_DifferenceEntropy |
|  | 238 | wavelet-LHH_glcm_DifferenceAverage |
|  | 239 | wavelet-LHH_glcm_Idn |
|  | 240 | wavelet-LHH_glcm_JointEnergy |
|  | 241 | wavelet-LHH_glcm_ClusterProminence |
|  | 242 | wavelet-LHH_glcm_InverseVariance |
|  | 243 | wavelet-LHH_glcm_ClusterTendency |
|  | 244 | wavelet-LHH_glcm_SumAverage |
|  | 245 | wavelet-LHH_glcm_Imc1 |
|  | 246 | wavelet-LHH_glcm_Contrast |
|  | 247 | wavelet-LHH_glszm_ZoneVariance |
|  | 248 | wavelet-LHH_glszm_GrayLevelVariance |
|  | 249 | wavelet-LHH_glszm_ZoneEntropy |
|  | 250 | wavelet-LHH_glszm_SizeZoneNonUniformity |
|  | 251 | wavelet-LHH_glszm_GrayLevelNonUniformity |
|  | 252 | wavelet-LHH_glszm_LargeAreaHighGrayLevelEmphasis |
|  | 253 | wavelet-LHH_glszm_ZonePercentage |
|  | 254 | wavelet-LHH_glszm_LargeAreaLowGrayLevelEmphasis |
|  | 255 | wavelet-LHH_glszm_SmallAreaHighGrayLevelEmphasis |
|  | 256 | wavelet-LHH_glszm_HighGrayLevelZoneEmphasis |
|  | 257 | wavelet-LHH_glszm_GrayLevelNonUniformityNormalized |
|  | 258 | wavelet-LHH_glszm_LowGrayLevelZoneEmphasis |
|  | 259 | wavelet-LHH_glszm_SizeZoneNonUniformityNormalized |
|  | 260 | wavelet-LHH_glszm_SmallAreaLowGrayLevelEmphasis |
|  | 261 | wavelet-LHH_glszm_LargeAreaEmphasis |
|  | 262 | wavelet-LHH_glszm_SmallAreaEmphasis |
|  | 263 | wavelet-LHH_firstorder_Kurtosis |
|  | 264 | wavelet-LHH_firstorder_TotalEnergy |
|  | 265 | wavelet-LHH_firstorder_Skewness |
|  | 266 | wavelet-LHH_firstorder_Median |
|  | 267 | wavelet-LHH_firstorder_InterquartileRange |
|  | 268 | wavelet-LHH_firstorder_10Percentile |
|  | 269 | wavelet-LHH_firstorder_RobustMeanAbsoluteDeviation |
|  | 270 | wavelet-LHH_firstorder_90Percentile |
|  | 271 | wavelet-LHH_firstorder_MeanAbsoluteDeviation |
|  | 272 | wavelet-LHH_firstorder_Variance |
|  | 273 | wavelet-LHH_firstorder_Uniformity |
|  | 274 | wavelet-LHH_firstorder_Maximum |
|  | 275 | wavelet-LHH_firstorder_Minimum |
|  | 276 | wavelet-LHH_firstorder_Mean |
|  | 277 | wavelet-LHH_firstorder_RootMeanSquared |
|  | 278 | wavelet-LHH_firstorder_Range |
|  | 279 | wavelet-LHH_firstorder_Entropy |
|  | 280 | wavelet-LHH_firstorder_Energy |
|  | 281 | wavelet-LHH_glrlm_GrayLevelVariance |
|  | 282 | wavelet-LHH_glrlm_LongRunLowGrayLevelEmphasis |
|  | 283 | wavelet-LHH_glrlm_RunPercentage |
|  | 284 | wavelet-LHH_glrlm_HighGrayLevelRunEmphasis |
|  | 285 | wavelet-LHH_glrlm_GrayLevelNonUniformity |
|  | 286 | wavelet-LHH_glrlm_ShortRunEmphasis |
|  | 287 | wavelet-LHH_glrlm_LowGrayLevelRunEmphasis |
|  | 288 | wavelet-LHH_glrlm_RunLengthNonUniformity |
|  | 289 | wavelet-LHH_glrlm_RunVariance |
|  | 290 | wavelet-LHH_glrlm_ShortRunLowGrayLevelEmphasis |
|  | 291 | wavelet-LHH_glrlm_LongRunEmphasis |
|  | 292 | wavelet-LHH_glrlm_ShortRunHighGrayLevelEmphasis |
|  | 293 | wavelet-LHH_glrlm_GrayLevelNonUniformityNormalized |
|  | 294 | wavelet-LHH_glrlm_LongRunHighGrayLevelEmphasis |
|  | 295 | wavelet-LHH_glrlm_RunLengthNonUniformityNormalized |
|  | 296 | wavelet-LHH_glrlm_RunEntropy |
|  | 297 | wavelet-HLH_glcm_MCC |
|  | 298 | wavelet-HLH_glcm_MaximumProbability |
|  | 299 | wavelet-HLH_glcm_Correlation |
|  | 300 | wavelet-HLH_glcm_Autocorrelation |
|  | 301 | wavelet-HLH_glcm_Id |
|  | 302 | wavelet-HLH_glcm_ClusterShade |
|  | 303 | wavelet-HLH_glcm_JointAverage |
|  | 304 | wavelet-HLH_glcm_Idm |
|  | 305 | wavelet-HLH_glcm_SumSquares |
|  | 306 | wavelet-HLH_glcm_SumEntropy |
|  | 307 | wavelet-HLH_glcm_Idmn |
|  | 308 | wavelet-HLH_glcm_JointEntropy |
|  | 309 | wavelet-HLH_glcm_DifferenceVariance |
|  | 310 | wavelet-HLH_glcm_Imc2 |
|  | 311 | wavelet-HLH_glcm_DifferenceEntropy |
|  | 312 | wavelet-HLH_glcm_DifferenceAverage |
|  | 313 | wavelet-HLH_glcm_Idn |
|  | 314 | wavelet-HLH_glcm_JointEnergy |
|  | 315 | wavelet-HLH_glcm_ClusterProminence |
|  | 316 | wavelet-HLH_glcm_InverseVariance |
|  | 317 | wavelet-HLH_glcm_ClusterTendency |
|  | 318 | wavelet-HLH_glcm_SumAverage |
|  | 319 | wavelet-HLH_glcm_Imc1 |
|  | 320 | wavelet-HLH_glcm_Contrast |
|  | 321 | wavelet-HLH_glszm_ZoneVariance |
|  | 322 | wavelet-HLH_glszm_GrayLevelVariance |
|  | 323 | wavelet-HLH_glszm_ZoneEntropy |
|  | 324 | wavelet-HLH_glszm_SizeZoneNonUniformity |
|  | 325 | wavelet-HLH_glszm_GrayLevelNonUniformity |
|  | 326 | wavelet-HLH_glszm_LargeAreaHighGrayLevelEmphasis |
|  | 327 | wavelet-HLH_glszm_ZonePercentage |
|  | 328 | wavelet-HLH_glszm_LargeAreaLowGrayLevelEmphasis |
|  | 329 | wavelet-HLH_glszm_SmallAreaHighGrayLevelEmphasis |
|  | 330 | wavelet-HLH_glszm_HighGrayLevelZoneEmphasis |
|  | 331 | wavelet-HLH_glszm_GrayLevelNonUniformityNormalized |
|  | 332 | wavelet-HLH_glszm_LowGrayLevelZoneEmphasis |
|  | 333 | wavelet-HLH_glszm_SizeZoneNonUniformityNormalized |
|  | 334 | wavelet-HLH_glszm_SmallAreaLowGrayLevelEmphasis |
|  | 335 | wavelet-HLH_glszm_LargeAreaEmphasis |
|  | 336 | wavelet-HLH_glszm_SmallAreaEmphasis |
|  | 337 | wavelet-HLH_firstorder_Kurtosis |
|  | 338 | wavelet-HLH_firstorder_TotalEnergy |
|  | 339 | wavelet-HLH_firstorder_Skewness |
|  | 340 | wavelet-HLH_firstorder_Median |
|  | 341 | wavelet-HLH_firstorder_InterquartileRange |
|  | 342 | wavelet-HLH_firstorder_10Percentile |
|  | 343 | wavelet-HLH_firstorder_RobustMeanAbsoluteDeviation |
|  | 344 | wavelet-HLH_firstorder_90Percentile |
|  | 345 | wavelet-HLH_firstorder_MeanAbsoluteDeviation |
|  | 346 | wavelet-HLH_firstorder_Variance |
|  | 347 | wavelet-HLH_firstorder_Uniformity |
|  | 348 | wavelet-HLH_firstorder_Maximum |
|  | 349 | wavelet-HLH_firstorder_Minimum |
|  | 350 | wavelet-HLH_firstorder_Mean |
|  | 351 | wavelet-HLH_firstorder_RootMeanSquared |
|  | 352 | wavelet-HLH_firstorder_Range |
|  | 353 | wavelet-HLH_firstorder_Entropy |
|  | 354 | wavelet-HLH_firstorder_Energy |
|  | 355 | wavelet-HLH_glrlm_GrayLevelVariance |
|  | 356 | wavelet-HLH_glrlm_LongRunLowGrayLevelEmphasis |
|  | 357 | wavelet-HLH_glrlm_RunPercentage |
|  | 358 | wavelet-HLH_glrlm_HighGrayLevelRunEmphasis |
|  | 359 | wavelet-HLH_glrlm_GrayLevelNonUniformity |
|  | 360 | wavelet-HLH_glrlm_ShortRunEmphasis |
|  | 361 | wavelet-HLH_glrlm_LowGrayLevelRunEmphasis |
|  | 362 | wavelet-HLH_glrlm_RunLengthNonUniformity |
|  | 363 | wavelet-HLH_glrlm_RunVariance |
|  | 364 | wavelet-HLH_glrlm_ShortRunLowGrayLevelEmphasis |
|  | 365 | wavelet-HLH_glrlm_LongRunEmphasis |
|  | 366 | wavelet-HLH_glrlm_ShortRunHighGrayLevelEmphasis |
|  | 367 | wavelet-HLH_glrlm_GrayLevelNonUniformityNormalized |
|  | 368 | wavelet-HLH_glrlm_LongRunHighGrayLevelEmphasis |
|  | 369 | wavelet-HLH_glrlm_RunLengthNonUniformityNormalized |
|  | 370 | wavelet-HLH_glrlm_RunEntropy |
|  | 371 | wavelet-HHL_glcm_MCC |
|  | 372 | wavelet-HHL_glcm_MaximumProbability |
|  | 373 | wavelet-HHL_glcm_Correlation |
|  | 374 | wavelet-HHL_glcm_Autocorrelation |
|  | 375 | wavelet-HHL_glcm_Id |
|  | 376 | wavelet-HHL_glcm_ClusterShade |
|  | 377 | wavelet-HHL_glcm_JointAverage |
|  | 378 | wavelet-HHL_glcm_Idm |
|  | 379 | wavelet-HHL_glcm_SumSquares |
|  | 380 | wavelet-HHL_glcm_SumEntropy |
|  | 381 | wavelet-HHL_glcm_Idmn |
|  | 382 | wavelet-HHL_glcm_JointEntropy |
|  | 383 | wavelet-HHL_glcm_DifferenceVariance |
|  | 384 | wavelet-HHL_glcm_Imc2 |
|  | 385 | wavelet-HHL_glcm_DifferenceEntropy |
|  | 386 | wavelet-HHL_glcm_DifferenceAverage |
|  | 387 | wavelet-HHL_glcm_Idn |
|  | 388 | wavelet-HHL_glcm_JointEnergy |
|  | 389 | wavelet-HHL_glcm_ClusterProminence |
|  | 390 | wavelet-HHL_glcm_InverseVariance |
|  | 391 | wavelet-HHL_glcm_ClusterTendency |
|  | 392 | wavelet-HHL_glcm_SumAverage |
|  | 393 | wavelet-HHL_glcm_Imc1 |
|  | 394 | wavelet-HHL_glcm_Contrast |
|  | 395 | wavelet-HHL_glszm_ZoneVariance |
|  | 396 | wavelet-HHL_glszm_GrayLevelVariance |
|  | 397 | wavelet-HHL_glszm_ZoneEntropy |
|  | 398 | wavelet-HHL_glszm_SizeZoneNonUniformity |
|  | 399 | wavelet-HHL_glszm_GrayLevelNonUniformity |
|  | 400 | wavelet-HHL_glszm_LargeAreaHighGrayLevelEmphasis |
|  | 401 | wavelet-HHL_glszm_ZonePercentage |
|  | 402 | wavelet-HHL_glszm_LargeAreaLowGrayLevelEmphasis |
|  | 403 | wavelet-HHL_glszm_SmallAreaHighGrayLevelEmphasis |
|  | 404 | wavelet-HHL_glszm_HighGrayLevelZoneEmphasis |
|  | 405 | wavelet-HHL_glszm_GrayLevelNonUniformityNormalized |
|  | 406 | wavelet-HHL_glszm_LowGrayLevelZoneEmphasis |
|  | 407 | wavelet-HHL_glszm_SizeZoneNonUniformityNormalized |
|  | 408 | wavelet-HHL_glszm_SmallAreaLowGrayLevelEmphasis |
|  | 409 | wavelet-HHL_glszm_LargeAreaEmphasis |
|  | 410 | wavelet-HHL_glszm_SmallAreaEmphasis |
|  | 411 | wavelet-HHL_firstorder_Kurtosis |
|  | 412 | wavelet-HHL_firstorder_TotalEnergy |
|  | 413 | wavelet-HHL_firstorder_Skewness |
|  | 414 | wavelet-HHL_firstorder_Median |
|  | 415 | wavelet-HHL_firstorder_InterquartileRange |
|  | 416 | wavelet-HHL_firstorder_10Percentile |
|  | 417 | wavelet-HHL_firstorder_RobustMeanAbsoluteDeviation |
|  | 418 | wavelet-HHL_firstorder_90Percentile |
|  | 419 | wavelet-HHL_firstorder_MeanAbsoluteDeviation |
|  | 420 | wavelet-HHL_firstorder_Variance |
|  | 421 | wavelet-HHL_firstorder_Uniformity |
|  | 422 | wavelet-HHL_firstorder_Maximum |
|  | 423 | wavelet-HHL_firstorder_Minimum |
|  | 424 | wavelet-HHL_firstorder_Mean |
|  | 425 | wavelet-HHL_firstorder_RootMeanSquared |
|  | 426 | wavelet-HHL_firstorder_Range |
|  | 427 | wavelet-HHL_firstorder_Entropy |
|  | 428 | wavelet-HHL_firstorder_Energy |
|  | 429 | wavelet-HHL_glrlm_GrayLevelVariance |
|  | 430 | wavelet-HHL_glrlm_LongRunLowGrayLevelEmphasis |
|  | 431 | wavelet-HHL_glrlm_RunPercentage |
|  | 432 | wavelet-HHL_glrlm_HighGrayLevelRunEmphasis |
|  | 433 | wavelet-HHL_glrlm_GrayLevelNonUniformity |
|  | 434 | wavelet-HHL_glrlm_ShortRunEmphasis |
|  | 435 | wavelet-HHL_glrlm_LowGrayLevelRunEmphasis |
|  | 436 | wavelet-HHL_glrlm_RunLengthNonUniformity |
|  | 437 | wavelet-HHL_glrlm_RunVariance |
|  | 438 | wavelet-HHL_glrlm_ShortRunLowGrayLevelEmphasis |
|  | 439 | wavelet-HHL_glrlm_LongRunEmphasis |
|  | 440 | wavelet-HHL_glrlm_ShortRunHighGrayLevelEmphasis |
|  | 441 | wavelet-HHL_glrlm_GrayLevelNonUniformityNormalized |
|  | 442 | wavelet-HHL_glrlm_LongRunHighGrayLevelEmphasis |
|  | 443 | wavelet-HHL_glrlm_RunLengthNonUniformityNormalized |
|  | 444 | wavelet-HHL_glrlm_RunEntropy |
|  | 445 | wavelet-LHL_glcm_MCC |
|  | 446 | wavelet-LHL_glcm_MaximumProbability |
|  | 447 | wavelet-LHL_glcm_Correlation |
|  | 448 | wavelet-LHL_glcm_Autocorrelation |
|  | 449 | wavelet-LHL_glcm_Id |
|  | 450 | wavelet-LHL_glcm_ClusterShade |
|  | 451 | wavelet-LHL_glcm_JointAverage |
|  | 452 | wavelet-LHL_glcm_Idm |
|  | 453 | wavelet-LHL_glcm_SumSquares |
|  | 454 | wavelet-LHL_glcm_SumEntropy |
|  | 455 | wavelet-LHL_glcm_Idmn |
|  | 456 | wavelet-LHL_glcm_JointEntropy |
|  | 457 | wavelet-LHL_glcm_DifferenceVariance |
|  | 458 | wavelet-LHL_glcm_Imc2 |
|  | 459 | wavelet-LHL_glcm_DifferenceEntropy |
|  | 460 | wavelet-LHL_glcm_DifferenceAverage |
|  | 461 | wavelet-LHL_glcm_Idn |
|  | 462 | wavelet-LHL_glcm_JointEnergy |
|  | 463 | wavelet-LHL_glcm_ClusterProminence |
|  | 464 | wavelet-LHL_glcm_InverseVariance |
|  | 465 | wavelet-LHL_glcm_ClusterTendency |
|  | 466 | wavelet-LHL_glcm_SumAverage |
|  | 467 | wavelet-LHL_glcm_Imc1 |
|  | 468 | wavelet-LHL_glcm_Contrast |
|  | 469 | wavelet-LHL_glszm_ZoneVariance |
|  | 470 | wavelet-LHL_glszm_GrayLevelVariance |
|  | 471 | wavelet-LHL_glszm_ZoneEntropy |
|  | 472 | wavelet-LHL_glszm_SizeZoneNonUniformity |
|  | 473 | wavelet-LHL_glszm_GrayLevelNonUniformity |
|  | 474 | wavelet-LHL_glszm_LargeAreaHighGrayLevelEmphasis |
|  | 475 | wavelet-LHL_glszm_ZonePercentage |
|  | 476 | wavelet-LHL_glszm_LargeAreaLowGrayLevelEmphasis |
|  | 477 | wavelet-LHL_glszm_SmallAreaHighGrayLevelEmphasis |
|  | 478 | wavelet-LHL_glszm_HighGrayLevelZoneEmphasis |
|  | 479 | wavelet-LHL_glszm_GrayLevelNonUniformityNormalized |
|  | 480 | wavelet-LHL_glszm_LowGrayLevelZoneEmphasis |
|  | 481 | wavelet-LHL_glszm_SizeZoneNonUniformityNormalized |
|  | 482 | wavelet-LHL_glszm_SmallAreaLowGrayLevelEmphasis |
|  | 483 | wavelet-LHL_glszm_LargeAreaEmphasis |
|  | 484 | wavelet-LHL_glszm_SmallAreaEmphasis |
|  | 485 | wavelet-LHL_firstorder_Kurtosis |
|  | 486 | wavelet-LHL_firstorder_TotalEnergy |
|  | 487 | wavelet-LHL_firstorder_Skewness |
|  | 488 | wavelet-LHL_firstorder_Median |
|  | 489 | wavelet-LHL_firstorder_InterquartileRange |
|  | 490 | wavelet-LHL_firstorder_10Percentile |
|  | 491 | wavelet-LHL_firstorder_RobustMeanAbsoluteDeviation |
|  | 492 | wavelet-LHL_firstorder_90Percentile |
|  | 493 | wavelet-LHL_firstorder_MeanAbsoluteDeviation |
|  | 494 | wavelet-LHL_firstorder_Variance |
|  | 495 | wavelet-LHL_firstorder_Uniformity |
|  | 496 | wavelet-LHL_firstorder_Maximum |
|  | 497 | wavelet-LHL_firstorder_Minimum |
|  | 498 | wavelet-LHL_firstorder_Mean |
|  | 499 | wavelet-LHL_firstorder_RootMeanSquared |
|  | 500 | wavelet-LHL_firstorder_Range |
|  | 501 | wavelet-LHL_firstorder_Entropy |
|  | 502 | wavelet-LHL_firstorder_Energy |
|  | 503 | wavelet-LHL_glrlm_GrayLevelVariance |
|  | 504 | wavelet-LHL_glrlm_LongRunLowGrayLevelEmphasis |
|  | 505 | wavelet-LHL_glrlm_RunPercentage |
|  | 506 | wavelet-LHL_glrlm_HighGrayLevelRunEmphasis |
|  | 507 | wavelet-LHL_glrlm_GrayLevelNonUniformity |
|  | 508 | wavelet-LHL_glrlm_ShortRunEmphasis |
|  | 509 | wavelet-LHL_glrlm_LowGrayLevelRunEmphasis |
|  | 510 | wavelet-LHL_glrlm_RunLengthNonUniformity |
|  | 511 | wavelet-LHL_glrlm_RunVariance |
|  | 512 | wavelet-LHL_glrlm_ShortRunLowGrayLevelEmphasis |
|  | 513 | wavelet-LHL_glrlm_LongRunEmphasis |
|  | 514 | wavelet-LHL_glrlm_ShortRunHighGrayLevelEmphasis |
|  | 515 | wavelet-LHL_glrlm_GrayLevelNonUniformityNormalized |
|  | 516 | wavelet-LHL_glrlm_LongRunHighGrayLevelEmphasis |
|  | 517 | wavelet-LHL_glrlm_RunLengthNonUniformityNormalized |
|  | 518 | wavelet-LHL_glrlm_RunEntropy |
|  | 519 | wavelet-LLL_glcm_MCC |
|  | 520 | wavelet-LLL_glcm_MaximumProbability |
|  | 521 | wavelet-LLL_glcm_Correlation |
|  | 522 | wavelet-LLL_glcm_Autocorrelation |
|  | 523 | wavelet-LLL_glcm_Id |
|  | 524 | wavelet-LLL_glcm_ClusterShade |
|  | 525 | wavelet-LLL_glcm_JointAverage |
|  | 526 | wavelet-LLL_glcm_Idm |
|  | 527 | wavelet-LLL_glcm_SumSquares |
|  | 528 | wavelet-LLL_glcm_SumEntropy |
|  | 529 | wavelet-LLL_glcm_Idmn |
|  | 530 | wavelet-LLL_glcm_JointEntropy |
|  | 531 | wavelet-LLL_glcm_DifferenceVariance |
|  | 532 | wavelet-LLL_glcm_Imc2 |
|  | 533 | wavelet-LLL_glcm_DifferenceEntropy |
|  | 534 | wavelet-LLL_glcm_DifferenceAverage |
|  | 535 | wavelet-LLL_glcm_Idn |
|  | 536 | wavelet-LLL_glcm_JointEnergy |
|  | 537 | wavelet-LLL_glcm_ClusterProminence |
|  | 538 | wavelet-LLL_glcm_InverseVariance |
|  | 539 | wavelet-LLL_glcm_ClusterTendency |
|  | 540 | wavelet-LLL_glcm_SumAverage |
|  | 541 | wavelet-LLL_glcm_Imc1 |
|  | 542 | wavelet-LLL_glcm_Contrast |
|  | 543 | wavelet-LLL_glszm_ZoneVariance |
|  | 544 | wavelet-LLL_glszm_GrayLevelVariance |
|  | 545 | wavelet-LLL_glszm_ZoneEntropy |
|  | 546 | wavelet-LLL_glszm_SizeZoneNonUniformity |
|  | 547 | wavelet-LLL_glszm_GrayLevelNonUniformity |
|  | 548 | wavelet-LLL_glszm_LargeAreaHighGrayLevelEmphasis |
|  | 549 | wavelet-LLL_glszm_ZonePercentage |
|  | 550 | wavelet-LLL_glszm_LargeAreaLowGrayLevelEmphasis |
|  | 551 | wavelet-LLL_glszm_SmallAreaHighGrayLevelEmphasis |
|  | 552 | wavelet-LLL_glszm_HighGrayLevelZoneEmphasis |
|  | 553 | wavelet-LLL_glszm_GrayLevelNonUniformityNormalized |
|  | 554 | wavelet-LLL_glszm_LowGrayLevelZoneEmphasis |
|  | 555 | wavelet-LLL_glszm_SizeZoneNonUniformityNormalized |
|  | 556 | wavelet-LLL_glszm_SmallAreaLowGrayLevelEmphasis |
|  | 557 | wavelet-LLL_glszm_LargeAreaEmphasis |
|  | 558 | wavelet-LLL_glszm_SmallAreaEmphasis |
|  | 559 | wavelet-LLL_firstorder_Kurtosis |
|  | 560 | wavelet-LLL_firstorder_TotalEnergy |
|  | 561 | wavelet-LLL_firstorder_Skewness |
|  | 562 | wavelet-LLL_firstorder_Median |
|  | 563 | wavelet-LLL_firstorder_InterquartileRange |
|  | 564 | wavelet-LLL_firstorder_10Percentile |
|  | 565 | wavelet-LLL_firstorder_RobustMeanAbsoluteDeviation |
|  | 566 | wavelet-LLL_firstorder_90Percentile |
|  | 567 | wavelet-LLL_firstorder_MeanAbsoluteDeviation |
|  | 568 | wavelet-LLL_firstorder_Variance |
|  | 569 | wavelet-LLL_firstorder_Uniformity |
|  | 570 | wavelet-LLL_firstorder_Maximum |
|  | 571 | wavelet-LLL_firstorder_Minimum |
|  | 572 | wavelet-LLL_firstorder_Mean |
|  | 573 | wavelet-LLL_firstorder_RootMeanSquared |
|  | 574 | wavelet-LLL_firstorder_Range |
|  | 575 | wavelet-LLL_firstorder_Entropy |
|  | 576 | wavelet-LLL_firstorder_Energy |
|  | 577 | wavelet-LLL_glrlm_GrayLevelVariance |
|  | 578 | wavelet-LLL_glrlm_LongRunLowGrayLevelEmphasis |
|  | 579 | wavelet-LLL_glrlm_RunPercentage |
|  | 580 | wavelet-LLL_glrlm_HighGrayLevelRunEmphasis |
|  | 581 | wavelet-LLL_glrlm_GrayLevelNonUniformity |
|  | 582 | wavelet-LLL_glrlm_ShortRunEmphasis |
|  | 583 | wavelet-LLL_glrlm_LowGrayLevelRunEmphasis |
|  | 584 | wavelet-LLL_glrlm_RunLengthNonUniformity |
|  | 585 | wavelet-LLL_glrlm_RunVariance |
|  | 586 | wavelet-LLL_glrlm_ShortRunLowGrayLevelEmphasis |
|  | 587 | wavelet-LLL_glrlm_LongRunEmphasis |
|  | 588 | wavelet-LLL_glrlm_ShortRunHighGrayLevelEmphasis |
|  | 589 | wavelet-LLL_glrlm_GrayLevelNonUniformityNormalized |
|  | 590 | wavelet-LLL_glrlm_LongRunHighGrayLevelEmphasis |
|  | 591 | wavelet-LLL_glrlm_RunLengthNonUniformityNormalized |
|  | 592 | wavelet-LLL_glrlm_RunEntropy |

GLCM = gray level cooccurrence matrix; GLRML = gray level run length matrix; GLSZM = gray level size zone matrix; SD = standard deviation.

Supplemental Table 2. Summary of top selected features and the estimates of coefficients by LASSO from results of cross validation

| Features | Selected Times | Estimate of Coefficient (Mean ± SD) |
| --- | --- | --- |
| DES-CC-wavelet.LLL_glcm_Imc2 | 490 | 0.5341 ± 0.0984 |
| LE-MLO-wavelet.HLH_glszm_ZoneEntropy | 485 | 0.5320 ± 0.1373 |
| HE-MLO-wavelet.HLH_glszm_ZoneEntropy | 468 | 0.2257 ± 0.1286 |
| LE-CC-wavelet.LLL_firstorder_InterquartileRange | 448 | 0.2371 ± 0.1104 |
| DES-MLO-wavelet.LLH_firstorder_Maximum | 365 | 0.1221 ± 0.0820 |
| LE-MLO-wavelet.LLL_firstorder_InterquartileRange | 242 | 0.1023 ± 0.0886 |
| HE-MLO-wavelet.LHH_glszm_ZoneEntropy | 228 | 0.0763 ± 0.0637 |
| DES-CC-wavelet.HHH_glszm_GrayLevelVariance | 221 | 0.0736 ± 0.0646 |
| DES-MLO-wavelet.LLH_glrlm_ShortRunHighGrayLevelEmphasis | 220 | 0.0921 ± 0.0770 |
| DES-MLO-wavelet.LHH_glszm_ZoneEntropy | 191 | 0.0670 ± 0.0560 |
| LE-MLO-wavelet.HHH_glszm_GrayLevelNonUniformity | 163 | 0.0922 ± 0.0785 |
| HE-MLO-wavelet.HHH_firstorder_Maximum | 147 | 0.0639 ± 0.0523 |

The features selected ≥ 20% times by LASSO in the cross validation were summarized.

Mean and standard deviation of estimate of coefficient were calculated when the feature was selected.

Supplemental Table 3. Summary of top 20 importance features and the permutation importance from RF from results of cross validation

| Features | Permutation Importance (Mean± SD) |
| --- | --- |
| LE-MLO-wavelet.HLH_glszm_ZoneEntropy | 0.0058 ± 0.0019 |
| LE-MLO-wavelet.HHH_glszm_GrayLevelNonUniformity | 0.0029 ± 0.0013 |
| DES-MLO-wavelet.LLL_glrlm_GrayLevelNonUniformity | 0.0023 ± 0.0010 |
| DES-CC-wavelet.LLL_glcm_Imc2 | 0.0022 ± 0.0011 |
| LE-CC-wavelet.LHH_glszm_ZoneEntropy | 0.0022 ± 0.0011 |
| DES-MLO-wavelet.LLH_glszm_SmallAreaHighGrayLevelEmphasis | 0.0022 ± 0.0010 |
| DES-MLO-wavelet.LLH_firstorder_Maximum | 0.0022 ± 0.0012 |
| DES-MLO-wavelet.HLL_firstorder_TotalEnergy | 0.0020 ± 0.0009 |
| LE-CC-wavelet.LLL_firstorder_InterquartileRange | 0.0019 ± 0.0010 |
| HE-CC-wavelet.LLL_glcm_Imc2 | 0.0018 ± 0.0010 |
| HE-MLO-wavelet.HLH_glszm_ZoneEntropy | 0.0015 ± 0.0009 |
| DES-MLO-wavelet.HHH_glrlm_GrayLevelNonUniformityNormalized | 0.0014 ± 0.0008 |
| LE-MLO-wavelet.LLL_firstorder_InterquartileRange | 0.0014 ± 0.0008 |
| DES-MLO-original_shape_Maximum3DDiameter | 0.0013 ± 0.0007 |
| LE-CC-wavelet.LLL_glcm_ClusterTendency | 0.0012 ± 0.0008 |
| LE-MLO-wavelet.LLL_glcm_Imc2 | 0.0012 ± 0.0007 |
| HE-CC-wavelet.LLL_glszm_LargeAreaHighGrayLevelEmphasis | 0.0011 ± 0.0007 |
| LE-CC-wavelet.LLL_glcm_MCC | 0.0011 ± 0.0008 |
| LE-CC-wavelet.LLL_glrlm_LongRunHighGrayLevelEmphasis | 0.0011 ± 0.0007 |
| DES-MLO-wavelet.LLH_firstorder_10Percentile | 0.0011 ± 0.0006 |

The features with highest permutation importance (top 20) were summarized.

Supplemental Table 4. Summary of image features and objective quantitative features in subgroups-of-interest based on LASSO regression models

| Image features | Category | Benign lesions | | p-value | Malignant lesions | | p-value |
| --- | --- | --- | --- | --- | --- | --- | --- |
|  |  | Misclassified  (n = 5) | Non-misclassified  (n = 37) |  | Misclassified  (n = 15) | Non-misclassified  (n = 79) |  |
| Lesion size ^*^ | / | 31.3 ± 11.8 | 14.9 ± 8.8 | 0.0003 | 19.5 ± 7.6 | 32.2 ± 17.0 | 0.0018 |
| Breast density | a-b | 1/5 (40.0) | 3/37 (8.1) | 0.3941 | 3/15 (20.0) | 25/79 (31.6) | 0.5403 |
|  | c-d | 4/5 (60.0) | 34/37 (91.8) |  | 12/15 (80.0) | 54/79 (68.4) |  |
| Degree of BPE | Minimal or mild | 1/5 (20.0) | 16/37 (43.2) | 0.6323 | 13/15 (86.7) | 59//79 (74.7) | 0.5076 |
|  | Moderate or marked | 4/5 (80.0) | 21/37 (56.8) |  | 2/15 (13.3) | 20/79 (25.3) |  |
| Rim artifact | Absent | 1/5 (20.0) | 36/37 (97.3) | 0.0001 | 14/15 (93.3) | 66/79 (83.5) | 0.4555 |
|  | Present | 4/5 (80.0) | 1/37 (2.7) |  | 1/15 (6.7) | 13/79 (16.4) |  |
| Ripple artifact | Absent | 3/5 (60.0) | 33/37 (89.2) | 0.1414 | 8/15 (53.3) | 41/79 (51.9) | 0.9999 |
|  | Present | 2/5 (40.0) | 4/37 (10.8) |  | 7/15 (46.7) | 38/79 (48.1) |  |
| Vascular artifact | Absent | 4/5 (80.0) | 32/37 (86.4) | 0.5568 | 11/15 (73.3) | 56/79 (70.9) | 1.000 |
|  | Present | 1/5 (20.0) | 5/37 (13.5) |  | 4/15 (26.7) | 23/79 (29.1) |  |
| Air trapping artifact | Absent | 5/5 (100.0) | 34/37 (92.3) | 0.9999 | 9/15 (60.0) | 67/79 (84.8) | 0.036 |
|  | Present | 0/5 (0.0) | 3/37 (7.7) |  | 6/15 (40.0) | 12/79 (15.2) |  |
| SNR ^*^ | / | 467.6 ± 100.8 | 154.2 ± 88.9 | < 0.0001 | 215.4 ± 89.6 | 406.2 ± 132.0 | < 0.0001 |
| CNR ^*^ | / | 539.7 ± 73.8 | 158.1 ± 96.3 | < 0.0001 | 237.8 ± 104.6 | 493.7 ± 180.5 | < 0.0001 |
| BCR ^*^ | / | 299.8 ± 38.4 | 88.2 ± 52.4 | 0.0003 | 131.8 ± 59.9 | 262.1 ± 98.4 | < 0.0001 |

^*^ Data are shown as mean values ± standard deviations. Other data are shown as proportions with percentages in parentheses.

LASSO = least absolute shrinkage and selection operator; BPE = background parenchymal enhancement; SNR = signal-to-noise ratio; CNR = contrast-to-noise ratio; BCR = background contrast ratio.

Supplemental Table 5. Multivariate factor analysis result in malignant lesions based on LASSO regression models.

| Image features | OR | 95% CI | p-value |
| --- | --- | --- | --- |
| Lesion size | 0.812 | (0.693, 0.905) | 0.0018 |
| Breast density (c-d) | 5.207 | (0.985, 41.667) | 0.0748 |
| Degree of BPE (moderate or marked) | 1.742 | (0.169, 16.670) | 0.6218 |
| Presence of rim artifact | 0.122 | (0.004, 1.518) | 0.1428 |
| Presence of vascular artifact | 0.964 | (0.153, 5.410) | 0.9672 |
| Presence of ripple artifact | 3.169 | (0.742, 15.531) | 0.1308 |
| Presence of air trapping artifact | 35.397 | (4.894, 458.956) | 0.0016 |

LASSO = least absolute shrinkage and selection operator; BPE = background parenchymal enhancement; OR = odds ratio; CI = confidence interval.

Supplemental Table 6. Summary of image features and objective quantitative features in subgroups-of-interest based on RF models.

| Image features | Category | Benign lesions | | p-value | Malignant lesions | | p-value |
| --- | --- | --- | --- | --- | --- | --- | --- |
|  |  | Misclassified  (n = 8) | Non-misclassified  (n = 35) |  | Misclassified  (n = 25) | Non- misclassified  (n = 81) |  |
| Lesion size ^*^ | / | 25.7 ± 12.7 | 15.3 ± 9.3 | 0.0156 | 16.7 ± 5.4 | 33.2 ± 16.1 | < 0.0001 |
| Breast density | a-b | 2/8 (25.0) | 2/35 (5.7) | 0.1514 | 4/25 (16.0) | 26/81 (32.1) | 0.1359 |
|  | c-d | 6/8 (75.0) | 33/35 (94.3) |  | 21/25 (84.0) | 55/81 (67.9) |  |
| Degree of BPE | Minimal or mild | 3/8 (37.5) | 15/35 (42.9) | 1.0000 | 19/25 (76.0) | 62/81 (76.5) | 1.000 |
|  | Moderate or marked | 5/8 (62.5) | 20/35 (57.1) |  | 6/25 (24.0) | 19/81 (23.5) |  |
| Rim artifact | Absent | 4/8 (50.0) | 34/35 (97.1) | 0.0026 | 23/25 (92.0) | 71/81 (87.7) | 0.7274 |
|  | Present | 4/8 (50.0) | 1/35 (2.9) |  | 2/25 (8.0) | 10/81 (12.3) |  |
| Ripple artifact | Absent | 3/8 (37.5) | 33/35 (85.7) | 0.0001 | 17/25 (68.0) | 39/81 (48.1) | 0.1092 |
|  | Present | 5/8 (62.5) | 2/35 (14.3) |  | 8/25 (32.0) | 42/81 (51.9) |  |
| Vascular artifact | Absent | 6/8 (75.0) | 32/35 (91.4) | 0.2276 | 21/25 (84.0) | 56/81 (69.1) | 0.2009 |
|  | Present | 2/8 (25.0) | 3/35 (8.6) |  | 4/25 (16.0) | 25/81 (30.9) |  |
| Air trapping artifact | Absent | 6/8 (75.0) | 34/35 (97.1) | 0.0840 | 19/25 (76.0) | 67/81 (82.7) | 0.5590 |
|  | Present | 2/8 (25.0) | 1/35 (2.9) |  | 6/25 (24.0) | 14/81 (17.2) |  |
| SNR2 ^*^ | / | 364.9 ± 167.0 | 137.1 ± 86.5 | 0.0002 | 267.7 ± 121.5 | 390.0 ± 131.7 | < 0.0001 |
| CNR ^*^ | / | 414.5 ± 187.8 | 142.3 ± 97.1 | 0.0001 | 299.6 ± 144.7 | 475.4 ± 183.6 | < 0.0001 |
| BCR ^*^ | / | 229.6 ± 105.8 | 79.8 ± 52.9 | 0.0009 | 160.4 ± 71.7 | 251.4 ± 95.2 | < 0.0001 |

^*^ Data are shown as mean values ± standard deviations. Other data are shown as proportions with percentages in parentheses.

RF = random forest; BPE = background parenchymal enhancement; SNR = signal-to-noise ratio; CNR = contrast-to-noise ratio; BCR = background contrast ratio.

Supplemental Table 7. Multivariate factor analysis result in malignant lesions based on RF models.

| Image features | OR | 95% CI | p-value |
| --- | --- | --- | --- |
| Lesion size | 0.713 | (0.592, 0.816) | < 0.0001 |
| Breast density (c-d) | 6.485 | (1.158, 52.097) | 0.0490 |
| Degree of BPE (moderate or marked) | 2.605 | (0.383, 20.844) | 0.3363 |
| Presence of rim artifact | 0.673 | (0.035, 9.591) | 0.7768 |
| Presence of vascular artifact | 0.288 | (0.0378, 1.656) | 0.1877 |
| Presence of ripple artifact | 0.899 | (0.207, 3.864) | 0.8848 |
| Presence of air trapping artifact | 15.148 | (2.179, 147.18) | 0.0097 |

RF = random forest; BPE = background parenchymal enhancement; OR = odds ratio; CI = confidence interval.
